# The architecture and effect of participation: a systematic review of community participation for communicable disease control and elimination. Implications for malaria elimination

**DOI:** 10.1186/1475-2875-10-225

**Published:** 2011-08-04

**Authors:** Jo-An Atkinson, Andrew Vallely, Lisa Fitzgerald, Maxine Whittaker, Marcel Tanner

**Affiliations:** 1 Pacific Malaria Initiative Support Centre, Australian Centre for International and Tropical Health, School of Population Health, University of Queensland, Brisbane, Australia; 2 The Kirby Institute (formerly National Centre in HIV Epidemiology and Clinical Research), University of New South Wales, Sydney, Australia & adjunct to School of Population Health, University of Queensland, Brisbane, Australia; 3 School of Population Health, University of Queensland, Brisbane, Australia; 4 Swiss Tropical and Public Health Institute, University of Basel, Basel, Switzerland & adjunct to School of Population Health, University of Queensland, Brisbane, Australia

## Abstract

**Background:**

Community engagement and participation has played a critical role in successful disease control and elimination campaigns in many countries. Despite this, its benefits for malaria control and elimination are yet to be fully realized. This may be due to a limited understanding of the influences on participation in developing countries as well as inadequate investment in infrastructure and resources to support sustainable community participation. This paper reports the findings of an atypical systematic review of 60 years of literature in order to arrive at a more comprehensive awareness of the constructs of participation for communicable disease control and elimination and provide guidance for the current malaria elimination campaign.

**Methods:**

Evidence derived from quantitative research was considered both independently and collectively with qualitative research papers and case reports. All papers included in the review were systematically coded using a pre-determined qualitative coding matrix that identified influences on community participation at the individual, household, community and government/civil society levels. Colour coding was also carried out to reflect the key primary health care period in which community participation programmes originated. These processes allowed exhaustive content analysis and synthesis of data in an attempt to realize conceptual development beyond that able to be achieved by individual empirical studies or case reports.

**Results:**

Of the 60 papers meeting the selection criteria, only four studies attempted to determine the effect of community participation on disease transmission. Due to inherent differences in their design, interventions and outcome measures, results could not be compared. However, these studies showed statistically significant reductions in disease incidence or prevalence using various forms of community participation. The use of locally selected volunteers provided with adequate training, supervision and resources are common and important elements of the success of the interventions in these studies. In addition, qualitative synthesis of all 60 papers elucidates the complex architecture of community participation for communicable disease control and elimination which is presented herein.

**Conclusions:**

The current global malaria elimination campaign calls for a health systems strengthening approach to provide an enabling environment for programmes in developing countries. In order to realize the benefits of this approach it is vital to provide adequate investment in the 'people' component of health systems and understand the multi-level factors that influence their participation. The challenges of strengthening this component of health systems are discussed, as is the importance of ensuring that current global malaria elimination efforts do not derail renewed momentum towards the comprehensive primary health care approach. It is recommended that the application of the results of this systematic review be considered for other diseases of poverty in order to harmonize efforts at building 'competent communities' for communicable disease control and optimising health system effectiveness.

## Background

Inspired by the successes being achieved with the campaign to eradicate smallpox, the World Health Organization (WHO) in the mid 1950s launched the Global Malaria Eradication Campaign. The focus of the campaign was interruption of the malaria parasite's transmission cycle through case detection and treatment as well as vector control, primarily with the use of a newly developed insecticide dicholoro-dephenyl-trichloroethane (DDT) [1]. The eradication initiatives introduced had considerable political and financial support and were launched simultaneously around the world with the exclusion of Africa. Eradication teams were deployed to spray millions of homes, dust forests and fields and drain wetlands in the vicinity of human settlements [1]. The WHO provided financial and technical support to assist countries in preparing comprehensive action plans, training personnel, implementation, monitoring and evaluation, and there was collaboration and coordination with international assistance agencies such as the United Nations Children's Fund (UNICEF), USAID and The Rockefeller Foundation [2].

As a result, malaria was eliminated from the US, Japan, Korea, Taiwan, Spain, Italy, the Balkans, Greece, northern Africa and parts of the South Pacific [3]. Countries that were successful in becoming malaria free were primarily those who had strong and advanced malaria control programmes prior to the commencement of the global eradication campaign [2]. Nevertheless, in the 1950s and 1960s, significant control was also achieved in countries with a history of meso-, hyper- and holoendemic malaria such as Sri Lanka, India and in the south-west Pacific [3-5].

Despite the promise these successes showed, progress soon began to falter. With the emergence of insecticide-resistant vectors, drug-resistant parasites, technical problems (such as DDT shortages) and due to a number of human behavioural factors, enthusiasm waned and political and financial support dwindled as it became apparent that the global eradication attempt could not succeed [6]. In addition, armed conflict, economic downturns and complex emergencies, caused breakdowns in primary health services, a collapse in malaria control programmes and resurgence of the disease [7]. By 1969 the eradication campaign was abandoned by the WHO and replaced with an endorsement for malaria control [8].

The limitations of the approach taken by the Global Malaria Eradication Campaign of the 1950's and 60's included assumptions that malaria eradication could be achieved using a one-size-fits-all strategy rather than by tailoring interventions to local contexts and that early successes of the campaign obviated the need for epidemiological and anthropological research [9]. A realisation of these limitations contributed to a shift in focus to a Primary Health Care (PHC) strategy for global health policy as proposed by the WHO and UNICEF at the Alma Ata Conference in 1978 [10]. Primary Health Care was defined by the WHO as, "essential health care made universally accessible to individuals and families in the community by means acceptable to them, through their full participation and at a cost that the community and the country can afford" [11].

The cornerstone of Primary Health Care is community participation, the popularity of which is premised on the perceived benefits of:

• the creation of an enabling environment for public health interventions;

• health behaviour modification and reasoned action as a by-product of augmented community empowerment and resilience;

• improved efficiency, utilisation and sustainability of health services; and

• the harnessing of community capacity and resources to supplement limited allocations for health care [12].

Community engagement and participation has played a critical role in successful communicable disease control and elimination campaigns in many countries [13-19]. Examples include malaria elimination in Taiwan in the 1960s; the elimination of schistosomiasis in Guangxi Province, China and malaria in Aneityum, Vanuatu in the 1990s; and elimination of onchocerciasis in 2002 in 11 West African Countries [14,17,19,20]. There are lessons to be garnered from current and historic examples of community participation, not least of which is, that the architecture of participation may vary significantly based on influences of factors including geographic location, disease impact, political context, economic conditions, resource availability and health policy.

The benefits of community participation for malaria control and elimination are yet to be fully realized. A study of community participation in 5 African countries in the programmes of the Roll Back Malaria Initiative, found the practical reality of community engagement in malaria control to be still generally low [21]. Possible explanations include; poor understanding of the constructs of participation in developing countries; inadequate health infrastructures and financial resources to support a community participation programme; and differing interpretations of the concept between policy makers, planners and health care professionals [21,22]. In addition, obtaining community support and enthusiasm for participation in intensified control and elimination activities in the context of disappearing disease, and maintaining it during the pre-elimination and surveillance phases of a programme, will be significantly more challenging than eliciting participation in an endemic or hyper-endemic context [23].

### Defining community participation

Difficulties with designing and implementing community participation programmes have in part been attributed to a lack of consensus on what constitutes 'community' and 'participation' [24]. A number of 'ladders' of participation have been presented in the literature since the late 1960s that theoretically define participation on the basis of the level of power citizens have in decision-making processes, however, a critique of these ladders is beyond the scope of this review [25-29]. Pragmatic geographical definitions of 'community' have dominated tropical disease control to date as they are consistent with the epidemiology of disease transmission, with vector ecology and environmental conditions influencing the vulnerability of people to infection [30]. There are those that suggest this definition of 'community' may be adequate, particularly in rural areas where groups *'living in the same geographical area and sharing the same problems and resources....know one another and have a feeling of togetherness' *[31]. However, geographical proximity does not always equate to social cohesiveness and shared interests, particularly where there are imbalances in resource availability, cultural heterogeneity, ethnic tensions, itinerant populations or governance systems that promote individualism [30,32,33]. The movement of people as a result of globalization has resulted in a highly dynamic social tissue with decision-making occurring more at the household level rather than the community level, particularly in non-rural settings [34]. Divergence in interests within geographical boundaries can be particularly evident in contemporary urbanized and industrialized settings [30,35]. The mobilization of collective community action in such settings may be sub-optimal when programmes fail to identify all stakeholders and influential community members and when there exist conflicts of interest, communication difficulties and differing educational needs [33,36].

Although programmes for communicable disease control and elimination will continue to be targeted geographically based on epidemiological evidence of population vulnerability and intervention effectiveness; it has been suggested that participation of populations may be considerably enhanced by having the recipients of intended interventions define what they perceive as their 'communities' [33]. Accordingly, the development of theoretical concepts and 'etic' (externally derived) definitions of community as the basis of participation should be discouraged [33]. Such pre-defined models may not be relevant at the local level, they can be simplistic and problematic and often do not address the heterogeneity across rural, urban and sub-urban areas or between stable and transient populations [30,37]. It has been proposed that local 'actors' (including representatives of the poor and marginalized) be facilitated to map the framework, membership and boundaries of what they experience as their 'communities,' whether it be determined by economic, political, cultural, geographic or administrative groupings or through a shared sense of identity based of beliefs or actions [30,32,33,38]

Similarly, it has been advocated that communities be given the opportunity to define their idea of 'participation.' This definition may be influenced by community and stakeholder perceptions of existing and expected levels of participation, community priorities and interests and the acceptability of the implementation of participatory interventions [35,39]. Consultations with communities to define these concepts in the formative stages of community participation strategy design will be an important first step to generating genuine and sustainable participation to support selective communicable disease control and elimination programmes.

### Approaches to community participation

Two conceptually different approaches to community participation have been debated in the literature for decades and in more recent times, this has been moderated by those searching to find the middle ground [22,40,41]. To summarize briefly here, the vertical or 'top-down' approach entails centralized development of objectives and action plans for community participation by policy makers and professionals who then endeavour to convince communities to actively participate in their implementation. This approach has merits in terms of logistical efficiency in planning and coordinating implementation of large scale, disease selective, national programmes. It is argued, however, that this paternalistic approach of imposing interventions on communities and convincing them to participate for the greater good will lead to behavioural resistance that can jeopardize health programmes, particularly in an environment where the disease is accorded a low priority in the eyes of the community [42].

The horizontal or 'bottom-up' approach to community participation seeks to engage and support communities in identifying and prioritizing their own health concerns in order to democratically make decisions regarding resource allocation, which professionals and local authorities are then asked to support [22]. The process of developing individual and community empowerment through this 'bottom-up' approach to participation is valuable for creating positive and sustainable health behaviour change, however, it requires a slow and iterative process and the development of strong, interactive community infrastructures [43]. While this approach is desirable, it often lacks the institutional roots to be able to generate sufficient resources to support each community's objectives [31]. In addition, it is inefficient for rapid national scale-up of programmes and incompatible with selective disease control or elimination agendas, particularly those funded primarily through external donor agencies [44].

A combined approach has therefore been advocated that aims to reconcile the interim efficiency of a vertical approach required for large scale coordinated planning and implementation, with the longer term goal of a sustainable community driven programme [19,20,41,45]. Discourse regarding approaches to community participation also highlights the importance of considering whether the purpose of participation is either a means to an end (creation of an enabling environment for effective disease control) or as an end in itself (as a path to empowerment and the realization of the PHC philosophy of the right to 'Health for All') [30,33,46].

Despite the importance of understanding definitions and approaches to community participation, in order to replicate past successes and to realize its full potential for malaria elimination, a more comprehensive understanding of the constructs of participation is needed. Therefore, the purpose of this paper is to systematically review the evidence and thematically deconstruct case reports of community participation over the past 60 years in order to arrive at an understanding of the architecture of participation for communicable disease control and elimination and provide guidance for the design of community participation strategies for malaria elimination.

## Methods

### Search strategy

A review was conducted of published primary and secondary data sources including qualitative and quantitative research, case reports and programme evaluations that have documented the impact and lessons learned from community participation in communicable disease control and elimination programmes between 1950 and 2010. This review was limited to communicable disease examples of community participation to maintain validity in applying the outcomes to malaria elimination. Literature searches and bibliography reviews to identify relevant publications were carried out from inception to September, 2010. Databases reviewed were Medline, PubMED, EMBASE, Web of Knowledge, the Cochrane Database of Systematic Reviews and Google Scholar. Key terms used in the search strategy included; 'community participation OR social mobilization OR community mobilization OR community action' AND 'health OR disease OR malaria OR polio OR smallpox OR guinea worm OR schistosomiasis OR vector borne disease OR communicable disease' AND 'elimination OR eradication OR control.' Both American and English spellings of key search terms were used. The search was limited to English language publications.

### Study selection

**Figure 1:**
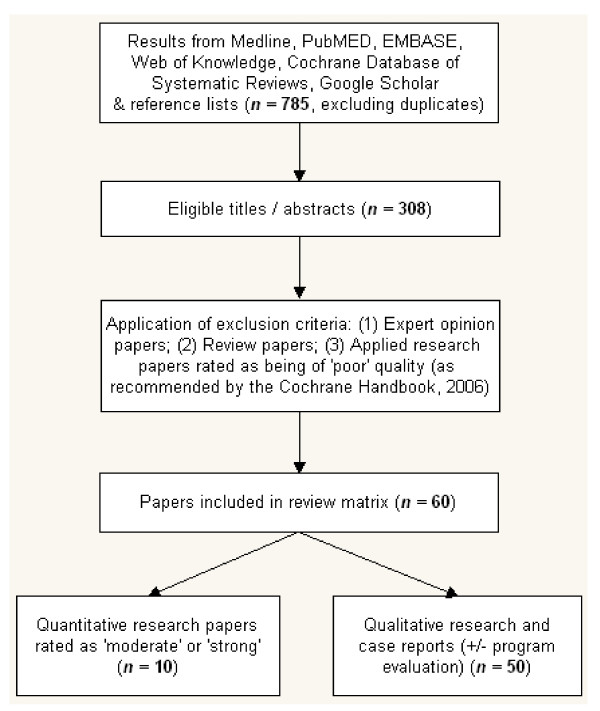
**Selection process from database search to final analysis**.

Papers eligible for inclusion were those reporting applied quantitative, qualitative or mixed method research rated as being of 'moderate' or 'strong' quality and investigating either the effect of community participation on communicable disease control or elimination; or the effect of the type of programme/strategy used on the level of participation achieved in the programme. In addition, case reports of community participation programmes including those with an evaluation component were also included in this review. Research rated as 'poor' quality, expert opinion papers and review papers were all excluded from the current review, although their reference lists were examined for relevant literature. The process of study selection is summarized in Figure 1. See Additional file 1 for summary of papers included in this review.

### Quality assessment, data coding and synthesis

In the absence of a 'gold standard' critical appraisal tool applicable across multiple study designs, the quality of quantitative research papers were defined as 'poor', 'moderate', or 'strong' based on standard epidemiological quality considerations relating to study design, data analysis and reporting as recommended by the Cochrane Handbook (2006) [47]. Evidence provided by applied research papers rated as 'moderate' or 'strong' was considered both independently and included in the coding framework.

**Figure 2:**
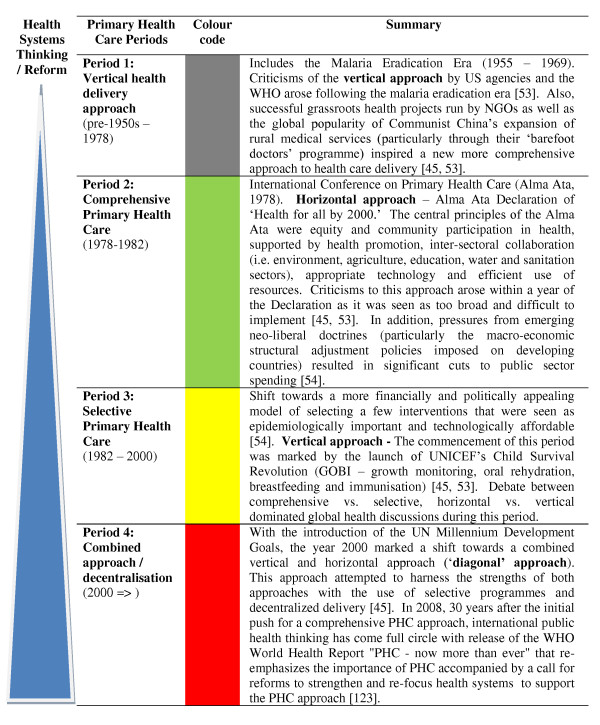
**Key periods of health systems development within the primary health care context**.

All papers included in the review were systematically coded using a pre-determined qualitative coding matrix that aimed to validate factors identified in review and expert opinion papers as influencing community participation at the individual, household, community and government/civil society levels (see Additional file 2). In addition, four key periods were identified where global health reform significantly altered approaches to health care delivery in the developing world. Each paper was, therefore, colour-coded to reflect the key PHC period in which community participation programmes originated in order to explore the linkage between international public health initiatives/approaches and the perceived role of communities within health systems (Figure 2).

The coding matrix allowed for analysis of differences in the influence of factors on community participation for communicable disease control versus elimination. Coded segments (mindful of their context) were then subject to exhaustive content analysis and synthesis in an attempt to realize conceptual development beyond that able to be achieved by individual empirical studies or case reports [48].

## Results

### Evidence for effectiveness of community participation

Of the 60 papers meeting the selection criteria, 12 reported findings of research on community participation. Four of these attempted to determine the effectiveness of community participation on disease control of which two were related to malaria control in Zaire and Malaysia, one to the prevention of sexually transmitted diseases in Nigerian youth, and one investigated the effectiveness of community participation for tuberculosis control in South Africa [49-52]. Due to inherent differences in their design, interventions and outcome measures, results cannot be compared. All studies however, showed a statistically significant reduction in disease incidence or prevalence using various forms of community participation outlined in Additional file 3. With varying levels of potential for bias and confounding found in these papers (ranging from moderate to low), the extent to which these reductions can be solely attributed to community participation is a matter for debate. Nevertheless, the use of locally selected volunteer health workers (or peer educators) provided with adequate training, supervision and resources are common and important elements of the success of the interventions outlined in these studies [49-52].

The remaining eight research papers examined the effectiveness of community participation strategies on the level of coverage and participation achieved. Again, differences in research design, interventions and outcome measures, prevent pooling of results, however, findings of each study are summarized in Additional file 3. Further lessons learned from the 12 research papers included in the review are detailed in Additional file 4.

**Figure 3:**
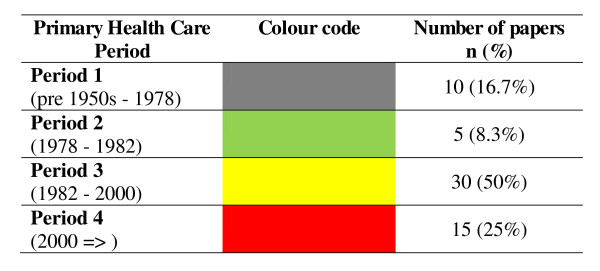
**Summary of the percentage of included papers outlining community participation that originated in each PHC period**.

Figure 3 summarizes the frequency of included papers occurring in each primary health care period. Although no comparative analyses can be made on this data, it is of interest that since the year 2000 there has been a marked fall in published literature outlining quality research or case reports on community participation for communicable disease control and elimination. This most likely reflects mainstream international public health thinking in the past decade which has seen global health initiatives that are primarily programme orientated and disease specific. Such initiatives favour vertical actions, are not principally systems-sensitive and often fail to view communities as components of health systems. In addition, the surprising paucity of papers on community participation arising from the period of comprehensive primary health care (Period 2) is most likely a consequence of the unpopularity of the approach in the immediate period following the Alma Ata Declaration. Viewed as too broad and difficult to implement and compounded by the effects of neo-liberal doctrines of macro-economic structural adjustment, comprehensive primary health care was soon replaced with the more financially and politically appealing model of selective primary health care [45,53,54].

Exhaustive content analysis of all 60 papers included in the review suggested that each of the proposed influencing factors listed in the coding matrix were addressed in varying degrees as having an impact on community participation (Table 1). Where there was sufficient heterogeneity in the influence of each factor between communicable disease control and elimination, these were presented separately; otherwise similar patterns were reported collectively.

**Table 1:** Factors influencing community participation and the percentage of included paper in which these factors were coded.

Participation	Influencing factors (proposed by literature referenced)	% of included papers in which factor was coded (number)
Individual level influences	Knowledge and perceptions of disease [34,130]	82% (49)
	
	Vulnerability versus resilience [30,95,131,132]	45% (27)
	
	Stigma [34,132]	15% (9)
	
	Incentives [132,133]	72% (43)
	
	Acceptability of intervention or programme [134]	60% (36)

Household level influences	Gender roles & power relationships [34,135]	22% (13)
	
	Cultural norms & social mechanisms [24,30,34]	45% (28)
	
	Access [34,136]	70% (42)
	
	Urban versus rural implementation [30]	10% (6)

Community level influences	Community characteristics [24,30,95,130,132,133,137]	43% (26)
	
	Disease epidemiology and complexity of intervention [130]	40% (24)
	
	Process by which communities are engaged to participate [24,30,137]	63% (38)
	
	Congruence of external targets and local priorities [30]	52% (31)

Government/civil society level influences	Political environment [30,132]	13% (8)
	
	Government advocacy and support [24,30,130,132,137]	47% (28)
	
	Health authority commitment to primary health care [30,130]	50% (30)
	
	Decentralisation of power and resources & use of community assets [24,30,130,132]	82% (49)
	
	Multisectoral collaboration and integration of programme into broader development goals [30,130]	67% (40)
	
	Financial and human resources [24,130]	82% (49)
	
	Techno-financial support & implementation style of locally embedded development agencies [137]	22% (13)

### Individual level determinants of participation

#### Knowledge and perceptions of disease

There was agreement across the literature on the influence of knowledge, perceptions and misconceptions of a disease on individual participation in preventative and treatment practices. Consequently, health education was recognized as the foundation of any community participation programme, particularly for its mitigating effect on barriers to participation including lack of perceived risk, stigma and intervention acceptability (to be discussed further).

##### Health education in a disease control context

Papers detailing communicable disease control programmes recognized disease knowledge and perceptions as significantly influencing participation and hence the design of locally-appropriate health education was considered a prerequisite for participation in the majority of these papers. The primary lessons learned from successes and failures of implementing health education as part of the community participation strategies in these control programmes are that:

• Bio-environmental and socio-economic influences on local transmission need to be understood by communities if they are to be empowered to effectively participate in programme planning and implementation. In addition, education should highlight the broader social and economic impact of the disease on communities and outline the benefits of participation in interventions or preventative measures [55,56].

• Prior to designing or re-assessing existing materials, preliminary investigations should be carried out to examine existing knowledge, folk beliefs and customs, current preventative and treatment-seeking practices (including the role of traditional healers), and acceptability of proposed interventions. The results of such investigations should inform the development of health messages that will address local issues and barriers to participation [56-61].

• Messages should be delivered regularly, through multiple established and respected networks (including schools, churches, mosques, community meetings, mass-media etc.) and should respect socio-cultural norms while challenging traditional beliefs [31,36,55-58,60,62-73].

• Inter-personal communication (via house-to-house visits) by respected community members/volunteers may be a more effective means of providing health education to elicit participation, particularly as it is interactive and provides access to information for remote or marginalized populations [62,65,71,73,74].

• Knowledge transfer through a mutual sharing of experiences between communities and programme staff allows a 'collective construction of knowledge.' This participatory approach to education that builds on indigenous knowledge is suggested to be more effective in facilitating community participation [33,67]. A study in Benin City, Nigeria investigating the effectiveness of a participatory approach through 'reproductive health clubs' and the use of peer educators found statistically significant reductions in the prevalence of STD symptoms using this model for behaviour change communication [51].

##### Health education in a disease elimination context

For programmes of disease elimination it was acknowledged that information alone is not enough [14,75-78]. Strategies for education depended on the nature and transmission of the disease. Taiwan's successful malaria elimination campaign included the provision of standardized guidelines, along with supporting IEC materials, to each health centre for the implementation of a publicity campaign in their townships during the immediate period preceding and following DDT spraying [14,77]. The successful schistosomiasis elimination campaign in Guangxi Province, China, erected warning placards at known snail breeding sites, sent worms to schools for display and went as far as carrying out live dissections of infected rabbits and mice at village exhibitions to demonstrate the worms and provide a visual component to messages that would leave a lasting impact [76]. In Bangladesh, during the campaign to eradicate smallpox, surveillance teams utilized the advantages of the disease being easily distinguishable and weekly markets at which every family was usually represented, to educate and question buyers and sellers using a picture of a patient with smallpox. This routine activity was reportedly responsible for the detection and containment of 80% of outbreaks in the country [79].

The temptation to shift from education to propaganda in the final push towards elimination can jeopardize sustainability of disease knowledge and preventative actions, hence papers highlighted the importance of persistent and comprehensive education programmes for success in achieving and sustaining motivation by maintaining the spotlight on the public health problem [76,78-80]. A multi-media, multi-channel approach was reported by most papers as having been vital for achieving sufficient coverage and impact of messages, particularly for resistant individuals and disadvantaged or isolated groups. Such media included the use of films, slides, radio, television, plays, songs, dances and days dedicated to disease elimination [76,78,80,81]. In addition, the schistosomiasis elimination campaign in China also invested in mass literacy classes with the aim of providing broader, long-term benefits for the health and economic development of communities [76].

**Figure 4:**
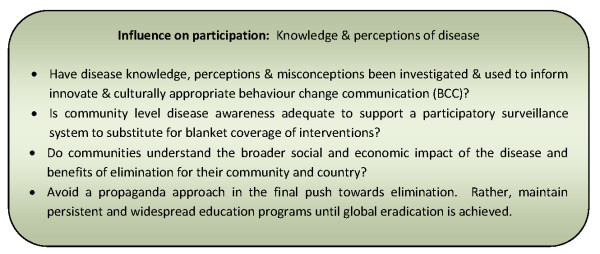
**Summary of considerations for community participation programmes relating to knowledge & perceptions of disease**.

Widespread disease awareness, with systems in place for timely reporting of cases or environmental risk factors, was advocated by a number of papers as allowing the effective implementation of a participatory surveillance system that provided a substitute for more costly blanket coverage of interventions [76,79,81,82]. This approach was identified as important throughout the phases of elimination, from intensified control through to 'holding the line' [76]. Figure 4 presents a summary of considerations relating to this influencing factor for community participation programmes.

#### Vulnerability versus resilience

**Figure 5:**
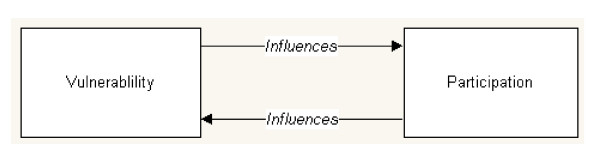
**Reciprocity of disease vulnerability and community participation**.

Almost half of the papers included in this review acknowledged the influence of vulnerability and resilience on participation. These papers elucidate the reciprocal relationship between disease vulnerability and participation (Figure 5).

Those that are vulnerable to communicable diseases due to biological and non-biological factors (such as age, reproductive status, poverty, political instability, education, access to services and livelihood conditions) are less capable of participating in disease prevention or control activities and hence become further vulnerable to disease [31,51,55,57,58,60,74,83-86]. When empowered to participate, however, populations take actions to mitigate risk and reduce their vulnerability to disease [31,51,55,58,60,61,63,65,71,84-91]. In doing so, individuals and communities can build capacity and resilience not only against selective diseases but more broadly in being able to advocate for their right to basic health services and engage in other agendas that influence community development [31,65,84,88,91].

Lack of a sense of ownership of a programme can affect receptiveness to health education, participation in preventative and curative measures, and can discourage discussion of issues and grievances [36]. Most papers, therefore, advocated that the most effective means of reducing vulnerability and achieving sustainable participation is to foster the building blocks of community resilience, namely, social identity, self-efficacy and empowerment. This was usually achieved by facilitating self-identification of problems in their community; and providing technical support to assist the design of their own solutions, and by doing so, tap into their latent capacity [36,55-58,60,84,87,89,92-94]. In effect, this approach describes the operationalization of Paolo Friere's notion of 'conscientization;' a process that sees individuals move from 'intransitive thought' or the belief that the conditions of life are out of one's control; to 'critical consciousness' where one is empowered through the use of critical thought and actions to change the conditions of life [89,95,96].

A community empowerment approach inspired by Friere's pedagogy has been used in Playa Municipality, Havana, Cuba since 2000 to encourage community participation in dengue control. At the outset, this programme saw communities themselves defining the concept of 'participation' and through a series of workshops, five participatory processes (capacity building, community surveillance, social communications, behaviour change and evaluation) were targeted to motivate and empower communities to take responsibility for managing vector control activities in their areas. An evaluation of this programme over six years found an improvement of participation in dengue prevention activities of more that 80% and a significant difference in entomological impact between the control and intervention areas throughout the study period [89].

As a further example, the malaria control programme in Indonesia in the 1980s introduced the concept of 'community self-survey,' a process of community identification, prioritization and planning of solutions for their own health needs which aimed to build community resilience. This approach reportedly contributed to sustainable community participation in the detection and treatment of malaria that exceeded expectations in the West Timor district [92].

The use of this approach is not necessarily limited to stable, peacetime contexts as was demonstrated in Tigray during the course of the Ethiopian civil war that spanned 17 years. Despite the instability and disease vulnerability created by civil war, the Tigrean People's Liberation Front were successful in achieving community self-reliance and participation in activities that promoted social and economic development, including the establishment of a PHC system and implementation of effective malaria control [84].

**Figure 6:**
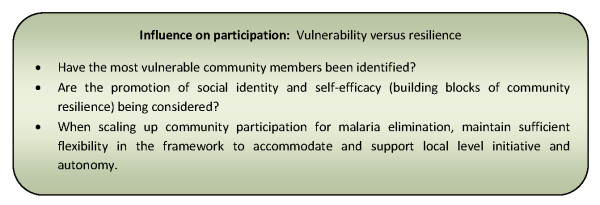
**Summary of considerations for community participation programmes relating to vulnerability versus resilience**.

Although advocated by many papers detailing disease control programmes, this approach was not reported in papers describing community participation for elimination programmes. This may be a result of outstanding questions of the scalability of the approach and how best to reconcile national level requirements for standardized implementation with the promotion of autonomy, initiative and empowerment at the local level [89]. Figure 6 presents a summary of considerations relating to this influencing factor for community participation programmes.

#### Stigma

Relatively few papers considered the issue of stigma of disease and its influence on participation. Although it was primarily described as a barrier to participation in treatment-seeking or case reporting, interestingly, it was also used as a motivation tool. While under British colonial rule, Cyprus succeeded in eliminating malaria through their intensive mosquito eradication campaign (1946-1950) [97]. A key component of this campaign was vector control, and groups of sanitary labourers lead by zone officers were assigned to areas to carry out larviciding activities. Each officer's work was regularly checked and the finding of *Anopheles *larva would result in the labelling of their designated area as 'dirty' [97]. Fear of stigmatization was used as a strategy to elicit high standards of participation from these groups of sanitary labourers.

Similarly in the 1980s, in an attempt build momentum for national participation in the eradication of guinea worm (an obscure and neglected disease in Nigeria at the time); a cover story detailing the shame and horror of this disease was published in local news magazine. This generated mass media attention to the problem which triggered a cascade of events that led to the launch of a national guinea worm eradication programme [80]. More commonly however, unless specifically addressed in programmes, stigma is a barrier to participation due to its psychological consequences and the social and economic isolation it can cause for those infected and their families [51,58,60,79,85,98].

**Figure 7:**
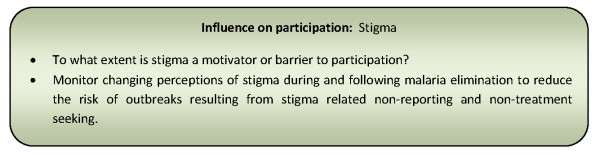
**Summary of considerations for community participation programmes relating to stigma**.

The broader impact of stigma was particularly evident during the smallpox eradication campaign [68]. Historically, in some cultures of West Africa, Asia and the Middle East, smallpox was attributed to the workings of the supernatural and often seen as an affliction brought on by the displeasure of the gods in human actions [79]. As a result, it has been estimated that over 95% of the world's smallpox cases went unreported in the pre-eradication era [79]. During the eradication campaign (1962-1979), the occurrence of outbreaks were seen by some health workers and officials to reflect poorly on regional or country vaccination performance and fears of dismissals or other reprisals resulted in adjustment of figures, suppression of reports and concealments of outbreaks [68]. In an attempt to counter such negative effects of stigma, a strategy of the Indian government was to introduce incentives and advocate the reporting of smallpox cases as a commendable action rather than one that might bring retribution. This facilitated early identification of outbreaks by health workers and members of the public [68,79]. Additional implications of incentives for community participation will be discussed in the following section. Figure 7 presents a summary of considerations relating to this influencing factor for community participation programmes.

#### Incentives

Almost three quarters of the papers included in this review made reference to intrinsic or extrinsic incentives as inducements for participation in communicable disease control or elimination. There was considerable diversity in the types of incentives provided across programmes and communities.

##### Control programme incentives

Remuneration for regular/full time work carried out in service of disease control programmes has been reported in some papers as a key element of their success in maintaining community participation, as it can provide a strong motivating influence and can underpin a mechanism of accountability [56,57]. However, the financial implications of meeting salaries for ongoing control programmes or lengthy elimination programmes can result in fewer than anticipated community-based workers being trained [99]. In addition, mismanagement of the distribution of cash incentives and selection of friends or relatives of community leaders for these positions can result in the recruitment of under-qualified workers [100]. Therefore, control programmes favour part-time or casual village-level volunteers to whom non-cash incentives are provided resulting in substantially more volunteers, but also higher attrition levels [99]. A study in South Africa on the use of lay volunteers for TB treatment delivery reported a gender differential with regards to demands for incentives and found that women were more willing to provide services on a volunteer basis without reward [52].

Non-monetary incentives for village-level volunteers have included personal development through training programmes, performance based rewards and promotions, positive publicity, free healthcare for their families, tax breaks, improved social status, and pride in the importance of their role in the success of a programme [31,50,67,83,84,92,98,100,101]. In addition, volunteer roles can be perceived as an avenue to employment [99]. Women in community volunteer roles have also reported increased support from their husbands, improved social standing and liberty to move about their localities that they were previously not afforded [31,99]. However, the reality of economic conditions in developing countries has made moral or intrinsic incentives for participation less desirable, which has led to increased requests to governments and health authorities for cash incentives [52,88,102].

Proponents of the Community Directed Intervention (CDI) approach for disease control have advocated a grassroots planning process that sees communities themselves design a sustainable incentive policy for motivating voluntary staff in keeping with local standards [91,94]. Furthermore, significantly less demands for monetary incentives have been achieved using a 'kinship enhanced' CDI approach that has community volunteers servicing only their kinships and hence less likely to make cash demands of their own relatives [36]. The CDI approach has the added benefit of shifting the perception of a programme from health authority to community responsibility [100]. Despite these potential benefits, parallel community-based development programmes that are providing remuneration to volunteers can undermine this process [94].

Other non-monetary incentives for participation reported include free health resources, and broader development benefits such as improvements in health, housing and other infrastructure, sanitation, education and income generation schemes [31,55,57,59,65,67,71,74,86,88,94,98,100,103,104]. Some programmes also reported conducting inter- school or community competitions that would popularize the project and provide basic but valued incentives such as stationary, clothing with programme logos or food items, which could be funded by NGO or private sector donations [67,105].

Using locally imbedded NGOs or other agencies to deliver incentives has been suggested as being particularly important for marginalized communities and those with strong traditional governance systems that are not easily enticed to participate in externally initiated programmes of which gains are seen as indirect [74]. Relationship building through the provision of incentives that address community priorities and investment in initiatives that improve the conditions and support their livelihoods of these communities will assist in motivating programme participation [74,106]. Conversely, donations and incentives that are perceived as short-term attempts to elicit participation with no genuine commitment to improving community health and development, will foster distrust and be damaging to current and future attempts at engaging communities [102].

A number of papers suggested that cash incentives are a lesser priority for empowered communities that are striving for self-reliance, since incentives are derived from the processes of engaging in political agendas, mobilizing assets and bringing about change for the benefit of their community [55,84,87,93]. With such diversity of cultures, needs and motivators across communities, even within a single country, remaining flexible, creative and perceptive to community motivators will be essential for providing a sustainable incentives system that maximizes participation [67].

##### Elimination programme incentives

Cash incentives have played a key role in eliciting and maintaining motivation for participation in disease elimination programmes both historically and in recent times [81]. Modest cash rewards were usually implemented to encouraged early treatment-seeking or underpin a participatory surveillance/case containment system and were often the driving force behind the detection of every last case of the disease, thereby breaking the transmission cycle [68,79,81]. In addition, cash incentives are particularly useful in achieving and maintaining the spotlight on a disease that has ceased to be a priority concern for communities [68,81].

In the Cameroon guinea worm elimination campaign, cash rewards were given to both the patient and the health worker only if the case was diagnosed within 24 hours of worm emergence. These rewards were funded in part from water filter sales and a donation from an international development agency. As cases decreased the value of cash rewards increased substantially and each village deemed to be actively participating in prevention activities received an additional cash payment. This incentive strategy and the community participation that it stimulated was reported to be a key determinant of the success of the programme [81]. A similar incentive programme was implemented during Taiwan's malaria elimination campaign with equal effect and contributed the sustained community surveillance and prevention of reintroduction of the disease [97]. If such incentive systems are to be introduced as part of a disease elimination programme, careful planning is required to prevent its exploitation through purposeful infection and to ensure mechanisms are in place for timely payment and publicity for those rewarded [81].

Economic incentives have been particularly influential in engaging and mobilizing national and provincial-level leaders in disease elimination programmes [80]. An economic impact study of guinea worm in south-eastern Nigeria in the late 1980s revealed a loss of $20 million per year which provided the initial momentum to embark on an elimination campaign [80]. The economic benefits of successful elimination of malaria in Cyprus was reported as being 17.6% annual return on the total expenditure of the campaign from savings on productive capacity and medical resources [97]. Mass participation of communities in environmental modification measures also provided economic incentives, with the increased availability of cultivable land boosting agricultural production [76,107].

**Figure 8:**
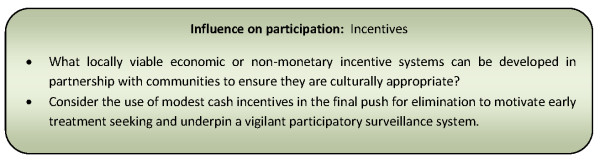
**Summary of considerations for community participation programmes relating to incentives**.

Other non-cash incentives to stimulate community participation in elimination programmes have included the provision of free diagnostic and curative services, distribution of free preventative interventions (such as bed nets or water filters), improvements in water quality and sanitation and food tokens in exchange for community labour [76,81,107,108]. Figure 8 presents a summary of considerations relating to this influencing factor for community participation programmes.

#### Acceptability of intervention or programme

Exogenous programmes and new intervention technologies can lack acceptability and have a profound impact on participation. In malaria control, it has been proposed that the lack of effectiveness of some programmes are a consequence of resistance having developed in one of three components of the transmission triangle, namely; physiological resistance of vectors, drug resistance by the parasites and 'human resistance' to interventions [103]. Discussions of the negative impact of poor acceptability on participation were not specific to interventions, diseases, programmes or regions.

In the Sarvodaya malaria control project in Sri Lanka in the 1980s, interventions such as repeated mass blood surveys and the use of the larvicide Monoxci^® ^in some communities had to be terminated due to their lack of acceptability [57]. In the early stages of the Cameroon guinea worm elimination campaign, lack of acceptability of the chemical treatment of drinking water sources (due to misconceptions and fear of the chemical) lead some community members to intentionally obstruct programme activities [81]. Factors affecting intervention acceptability reported in the papers reviewed are summarized in Table 2.

**Table 2:** Factors affecting acceptability of communicable disease control and elimination programmes identified in papers reviewed and ranked in order of extent of influence.

Factors affecting acceptability ranked in order of proposed degree of influence on participation	References
1. Lack of perceived risk of the disease	[104]

2. Inadequate knowledge about the reasons for and safety of the interventions	[58,61,78,84,90,98]

3. Inconsistency in service provision or doubts about the quality or usefulness of the service	[62,99,112]

4. Cost and side effects of interventions	[36,74,75,83]

5. Pervasive beliefs that the interventions have been introduced to intentionally cause harm or control populations (linked to abortion, infertility, brainwashing tool for communism)	[64,84]

6. Gender - i.e. acceptability of malaria services by women in a programme where almost all volunteers are men	[84]

7. Concerns about environmental contamination	[103]

8. Persistent fears of recurrence of previous colonial disease control practices such as house and body burnings.	[79]

In addition to these factors, previous control or elimination programme failures in resource poor settings can act to substantiate community misgivings of their health service and its lack of capacity and efficiency to deliver on the health needs of the people [52,88]. Determining and addressing such programme-level acceptability issues can be challenging. However, honesty and transparency regarding programme successes and challenges, creating awareness of intervention and implementation difficulties and the promotion of a community partnership approach in striving for solutions has been suggested to address issues of a lack of confidence in the programme [88]. Education, popularization and advocacy at all levels (national, district and community) was also used to increase acceptability of programmes and their activities [77,94].

In relation to intervention acceptability, time and the use of culturally appropriate and respected communication channels were suggested as important [55,56,60,74]. During the polio elimination campaign in India, failure to understand the rationale of vaccinations combined with misconceptions and suspicions as to the motive for the campaign in the absence of more pressing basic services, led to decreased vaccination acceptability and access in the most vulnerable communities [78]. Education was not enough for these underserved communities. To address this, village mobilization coordinators teamed with vaccination staff for routine follow-ups of families. Intensive and sustained engagement activities of religious leaders and influential community members combined with interpersonal communication (house-to-house visits) in underserved or resistant communities resulted in improved vaccination acceptability, fewer refusals, increased coverage and a subsequent decrease in polio incidence [78]. Similarly, intervention acceptability during the smallpox eradication campaign was aided by the recruitment of respected local volunteers to who were able to increase the acceptability of vaccinations by convincing them that the suffering and death caused by smallpox among their own people, after centuries of affliction, could finally be eliminated through participation in the campaign [79].

**Figure 9:**
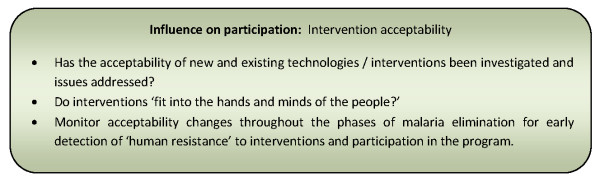
**Summary of considerations for community participation programmes relating to intervention acceptability**.

Other programmes addressed acceptability issues through forums for discussion to address concerns arising prior to and following implementation of interventions (including interaction with and consideration of product/intervention options where feasible) which assisted in allaying fears and increasing familiarity and acceptability of interventions [55,56,75]. In addition, a few papers reported the importance of regular monitoring of changing human perceptions and responses to interventions [57,75,78,106]. In Pakistan, epidemiological, social and behavioural research guided the design of behaviour change communication (BCC) strategies for targeted audiences which improved vaccination acceptability and participation in the polio elimination programme [78]. Such monitoring is suggested to achieve early detection of 'human resistance' arising from poor acceptability of interventions and programmes [75,106]. Finally, to facilitate acceptability, a number of papers advocated that interventions be effective, harmless, affordable, and should 'fit into the hands and minds of the people' [55,67,94,100,103]. Figure 9 presents a summary of considerations relating to this influencing factor for community participation programmes.

### Household level determinants of participation

#### Gender roles and power relationships

The influence of gender roles and power relationships on participation primarily focussed on women's capacity to act as community health volunteers. Many of these papers commented that traditional social systems usually place men in positions of power in the household and community, and women under restrictions that limit their influence and activity beyond their family [31,36,55,78,83,84]. However, the reported impact of these social norms and traditional gender roles on women's participation varied considerably for reasons unclear; possibly due to intermediary factors such as literacy and economic stability, or features of the programme.

Low participation of women in volunteer community health worker roles in the Tigray region, Ethiopia in the early to mid 1990's following the civil war was attributed to a combination of high illiteracy, significant domestic responsibilities and cultural norms [84]. In addition, their lack of access to knowledge about the importance of early treatment-seeking for malaria, lack of confidence in expressing needs to male decision-makers, and concerns regarding the perceptions of disloyalty when a woman seeks care from a male health volunteer resulted in inadequate treatment-seeking for fever. This resulted in an estimated 48% of children under 5 years with severe malaria dying without receiving care from a local health worker or facility [84].

In Thailand in the 1980's, transition from subsistence to a market-orientated economy resulted in further class and gender inequalities. As a result of limited and competitive employment opportunities in this new economy, men increasingly became the sole determinant of their family's economic situation, further disempowering women. This transition was suggested to have contributed to a reduction in community participation in prevention and control measures and a worsening malaria situation [83].

In contrast, disease control programmes in Sri Lanka, Guatemala and Kenya also with traditional social systems characterized by gender inequalities, all reported a majority of women at the heart of project activities and occupying roles of village volunteers [31,57,101]. Literacy was not a pre-requisite for selection as a village health volunteer in any of these programmes; however, literate, unemployed women were predominately selected for these roles [31,57,99,101].

**Figure 10:**
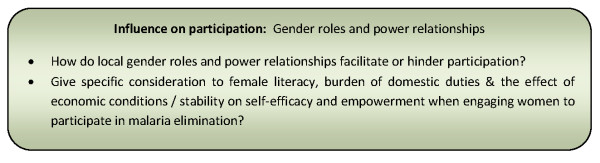
**Summary of considerations for community participation programmes relating to gender roles and power relationships**.

An emerging theme is the proposition that when communities are empowered with decision-making in disease control and elimination programmes they are more likely to negotiate roles for women that have previously been in conflict with their social norms [36,55]. This improved women's assertiveness in negotiating more active participation as they became increasingly aware of the importance of their role in the programme [36,91]. Traditional social systems characterized by gender inequalities are not necessarily a barrier to participation, however, issues such as female literacy, the burden of domestic duties, economic conditions and stability should be given specific consideration when attempting to engage women in disease control and elimination programmes [58,94]. Figure 10 presents a summary of considerations relating to these influencing factors for community participation programmes.

#### Consideration of cultural norms and social mechanisms

Almost half of the papers reviewed make reference to the importance of considering cultural norms and social mechanisms in the design and implementation of both community participation programmes and behaviour change communication (BCC) materials. A number of programmes suggested that in order to promote participation, the relevance, mode and style of delivery of disease control interventions and BCC materials need to be in harmony with the community's cultural experience and social routines [55,56,61,72,94,103,105]. In addition, the importance of early engagement of community opinion leaders and networks with representation from all ethnic, religious and social groups was important for ensuring that participation was inclusive [66,67,92,109].

Through human behavioural research, some disease control projects systematically explored cultural norms and practices and perceptions relevant to transmission which facilitated the exchange of 'exogenous' and 'indigenous' knowledge to enhance the cultural relevance and effectiveness of interventions [33,57,60,67]. Investigations into the background and functioning of existing social mechanisms have also been identified as important [60,74]. In Pondicherry, India, an attempt to mobilize youth for vector control activities through the formation of new 'youth clubs' failed as community leaders were afraid that youth from the lower income strata would become organized, operate outside their authority and rebel. These clubs were, therefore, disbanded to avoid the creation of civil tension [74].

Hence a valuable lesson learned from a number of programmes was that in order to effectively mobilize local communities, existing social or administrative mechanisms should be engaged rather than introducing new parallel structures [57,83]. No matter how democratically elected, power relationships of existing leadership systems tend to reproduce themselves, which can result in the failure of newly introduced committees or structures [31].

There was general consensus across the literature reviewed that households are best engaged through existing social mechanisms. Existing power structures and community organizations usually have well-established administrative hierarchies, additional resource capacity, links with linguistically and culturally diverse populations and a more intimate knowledge of areas and their problems [56,60,67,85,91,94,105]. They often have a history of promoting cooperative action within their communities and are therefore more viable as functioning groups that those established for the sole purpose of disease control or elimination [31,57,110]. Involving communities through social networks in the design and implementation of disease control and elimination programmes were also reported to have the added advantage of ensuring its legitimacy and sustainability [50,56,57,60,67,91].

**Figure 11:**
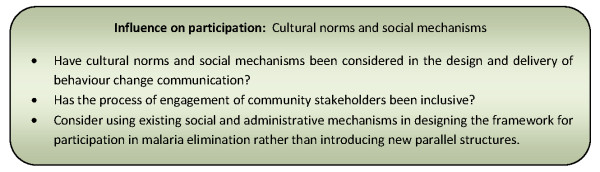
**Summary of considerations for community participation programmes relating to cultural norms and social mechanisms**.

Capitalizing on the influence of kinship systems has been suggested as an important motivator of household participation for tribal communities [36,55]. Engaging households through these systems can extend participation across both urban and rural communities [55]. The motivating effect may be due to often stronger affiliation of households with their extended family groupings than with any other community organizations [36,55]. Figure 11 presents a summary of considerations relating to these influencing factors for community participation programmes.

#### Access

Critical features of any disease elimination campaign are comprehensive coverage of preventative and curative interventions and broad, responsive surveillance systems [68,75,76,78,80,81]. Achieving access to poor, marginalized, remote and itinerant populations, however, has been a key stumbling block in number of disease elimination programmes [68,78,79]. Without access to and participation of these populations, pockets of disease transmission can remain and undermine progress [14,75,76,78-80]. In the papers reviewed, strategies to achieve access to these underserved populations varied depending on population density, the availability of human resources, and the ability to engage a sufficient range of socio-cultural opinion leaders [14,68,78,79]. For example, to achieve high participation in vaccination and surveillance activities during the smallpox eradication campaign, a systematic, house-to-house approach to education and vaccination was employed in rural and remote areas, whereas central vaccination points were used in densely populated areas with inclusive engagement of stakeholder representatives from vulnerable and marginalized populations [68].

Further lessons derived from disease control programmes are that access encompasses affordability, accessibility, adequacy and availability; and that its influence on household participation may be mediated by other potential participation determinants discussed previously such as education, vulnerability factors, stigma, acceptability, gender roles and power relationships.

Many papers reported the importance of affordability and convenience in improving access to and utilization of preventative and curative interventions [49,56,59,67,102,104]. The introduction of cost recovery systems, however, usually resulted in decreased participation [56,59,88,102]. In addition, activities that are demanding of time without any material incentive may also discourage participation as the primary concern of households is their livelihood and care of their family [74,88].

Geographical accessibility and adequacy of the local implementing infrastructure were also reported as important influences on participation [36,49,56,62,72,85,88,91,102,105]. Factors such remoteness, poor weather, availability of transport and inadequate road infrastructure can hamper implementation of prevention and control activities, supervision of health workers or volunteers and reduce the availability of vital health promotion and preventative and curative services [57,62,71,72,92]. In concurrence with findings related to stigma, social mechanisms and power relationships; socio-cultural accessibility was also identified as having an important impact on participation [36,60,74,85,90].

**Figure 12:**
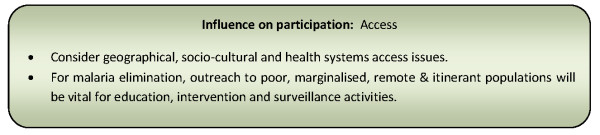
**Summary of considerations for community participation programmes relating to access**.

A number of strategies were used in disease control programmes to improve access and hence community participation. The introduction of additional village level volunteers, particularly those drawn from similar socio-cultural backgrounds to the populations they are serving, usually had the effect of increasing community access to health education and diagnostic and treatment services, and by doing so, increase household participation in the programme [31,36,49-51,57,58,91,92,94,98,100]. However, vulnerable groups were still found to under-utilize services provided by such volunteers due to barriers created by inadequate knowledge of or perceived risk of the disease or as a consequence of gender roles and power relationships [84,111]. Additional strategies suggested to improve access include the use of an empowerment approach to participation, harnessing 'kinship' systems and improving literacy [55,62,64,71]. Improvements in access to resources and services for disease control and elimination that produce tangible results can have a rousing effect on community confidence in the programme and stimulate proactive participation [65,91,103]. Figure 12 presents a summary of considerations relating to this influencing factor for community participation programmes.

#### Urban versus rural implementation

Although addressed in few papers, the degree of community participation reportedly differs between urban and rural populations. The dynamic social tissue within urban centres and between urban and rural areas created by economically stimulated rapid urban growth, increased population mobility and community heterogeneity and complicates efforts in achieving participation of urban communities [64,65,90,101]. In addition, urban communities are likely to rely more heavily on the private sector for their health needs than rural communities, and hence public sector health programmes can be less successful in engaging this population [90]. Strategies used to increase community participation in urban areas have therefore included; collaboration with the formal private sector; intensive profiling of urban communities prior to engagement activities to ensure representation of all stakeholder groups (including ethnic minorities, students and prisoners); the development of an urban area specific strategy for community participation; and capitalising on social linkages across urban and rural communities [55,65,90].

**Figure 13:**
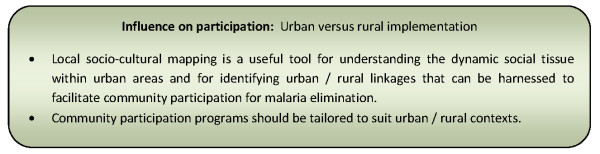
**Summary of considerations for community participation programmes relating to urban versus rural implementation**.

Mass emigration of men from rural areas to urban, industrial or mining areas can also lead to reduced capacity in rural areas for participation in disease prevention and control. This may be a consequence of the increased burden on those left behind, aspects of traditional life being in decline, reduced agricultural production, reduced nutritional status and increased vulnerability to disease [93]. Traditional kinship systems provide the opportunity for networking between urban and rural members [55]. Capitalizing on these social linkages may be an important strategy for breaking the poverty-vulnerability-disease cycle in some rural and remote communities through the ability of urban affiliates to influence their participation in disease prevention and control measures [55]. Figure 13 presents a summary of considerations relating to this influencing factor for community participation programmes.

### Community level determinants of participation

#### Community characteristics

Community characteristics may be determined by factors such as population size and mobility, age structure, income, geographic location, cultural diversity, leadership patterns, and importantly by a historic identity which may be shaped by indigenous, colonial and economic influences [64,65,74,77,93,102,112]. These characteristics are suggested to have a relationship to the degree and potential for community participation in communicable disease control [31,55,57,64,68,74,83,93,102,112].

Communities that are relatively homogenous in terms of ethnic, political, class, religious or kinship groupings are suggested to be more socially cohesive and willing to engage in collective and cooperative actions to meet their civil obligations [31,50,55,57]. In such environments, stable and adaptive social systems act to reduce the degree of inequality and fosters community spirit [57]. However, community participation programmes that have defined communities in geographical terms and erroneously assumed their homogeneity have been less successful in eliciting participation [33,83]. This is corollary to the additional complexity of community engagement, communication and education in heterogeneous communities [74,106]. The potential for participation has been limited by failures to identify all appropriate community representatives or stakeholders; not addressing varying levels of health education needs or communication channels; and having inadequate understanding of conflicts of interest, opposing political ideologies and group rivalries characterizing some communities [33,56,74].

As well as being heterogeneous, communities can be dynamic and tumultuous as a result of imposed political, economic and social transformation, which can profoundly impact participation in disease control [83]. For example, a traumatic history of civil war and genocide such as that experienced by Cambodians in the 1970s under autocratic rule of the Khmer Rouge has eroded community cohesion, cooperation and trust, undermining attempts at community participation long after the cessation of violence [102,110]. Further, economic modernization and commercialism can lead to poor social organization in peripheral communities as a consequence of strong centralising forces encouraging urbanization, selective education and economic emphasis on industrialization above agriculture [93].

Structural adjustment programmes introduced in developing countries in the 1980s and 1990s, lead to free-market policies and privatization in many sectors including health [76,113]. These dramatic economic changes can have a profound effect on community characteristics and the 'spirit of public service' through amplifications of class and gender inequalities, shift to autocratic leadership styles, breakdown of former commune structures and declining health and socio-economic conditions [83,108,113]. It is suggested that as a consequence of such economic policies, the post-modern era has seen a movement away from collectivism, to individualism and a focus on incentives to motivate participation in volunteerism due to the increased financial pressure on households to afford basic needs [76,88,107]. In addition, with traditional agrarian communities increasingly exposed to a cash economy and its stratifying effects, activities such as ploughing and harvesting that were once the basis of community cooperation, unity and a muting influence on economic and political inequalities, are now contracted out to itinerant workers [88]. The effects of such economic reform on communities will make participation in disease control and elimination more difficult to generate and sustain [24].

**Figure 14:**
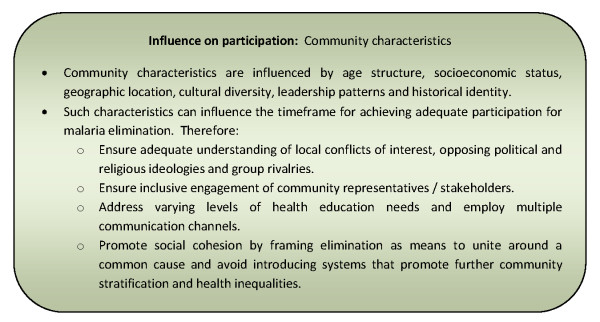
**Summary of considerations for community participation programmes relating to community characteristics**.

Counteracting these negative influences are community characteristics that contribute to successful collective community action for communicable disease control programmes. These include; a strong tradition of social participation, political mobilization or deep-rooted respect for leaders; religious endorsement of volunteerism or well-developed social and administrative structures [36,56,57,66,85,88,90,99,108-110]. Such community characteristics may have a considerable influence on the timeframe in which communities can be engaged and develop the capacity to design, implement and evaluate their participation in communicable disease control programmes [50,93]. Figure 14 presents a summary of considerations relating to this influencing factor for community participation programmes.

#### Disease epidemiology and complexity of interventions

Not all instances of successful community participation programmes have had a demonstrable or sustainable impact on disease incidence [57,72,104]. Mobilizing communities around use of the most effective tools is critical to success [57,103]. Therefore, the reality of most disease control or elimination programmes is that disease epidemiology and vector and intermediate host ecology often dictate the nature, distribution and complexity of interventions [14,50,61,62,74-76,80,97,98,100,101,109]. Given this reality, earlier importance given to intervention acceptability is further enhanced [61,74,75,88]. Although the complexity or demands of interventions are tolerated and motivation for participation more easily generated when in high transmission or outbreak prone areas [55,57,64,76]; this is not always the case [61,74,88]. To motivate participation at the community level, interventions should be within the capacity of communities to implement as well as provide notable and sustainable effects in reducing disease transmission [14,64,72,74,94,103].

Some communicable diseases (such as urinary schistosomiasis, onchocerciasis, guinea worm, and historically, smallpox) are more suited to community-based control and elimination efforts due to their distinctive symptoms, the availability of simple technology for rapid diagnosis and effective treatment, or prevention methods that are simple, practical and affordable to implement (with minimal support) at the community level [67-69,80,100,109]. Stratification and efficient implementation of interventions based on transmission intensity, vector or host density and intervention effectiveness also influence the complexity of interventions and nature of participation at the community level [14,50,76,80,83,101]. It is, therefore, imperative that strategies for community participation be tailored to meet the objectives of the control programme in partnership with communities in different epidemiological settings within countries [114].

**Figure 15:**
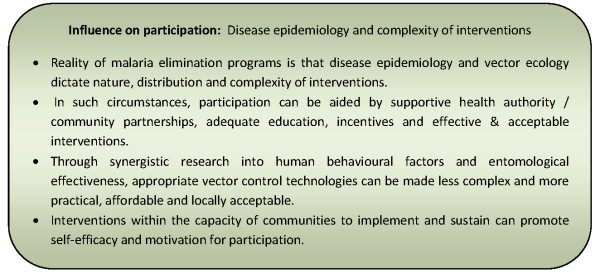
**Summary of considerations for community participation programmes relating to disease epidemiology and complexity of interventions**.

The credibility of community participatory strategies however, can be undermined by restricting participation to non-technical and labour-intensive interventions (such as environmental modification) as these can be perceived by communities as lacking efficacy and being poor quality substitutes to technical or chemical interventions [39]. Therefore, through synergistic research into human behavioural factors and entomological effectiveness, appropriate intervention technologies can be made less complex and more practical, affordable and locally acceptable in order to stimulate self-efficacy in their use and sustain participation [72,88]. Figure 15 presents a summary of considerations relating to this influencing factor for community participation programs.

#### Process by which communities are engaged to participate

The success and sustainability of community participation in health and development projects has been attributed to the extent to which community ownership and empowerment is achieved [31]. Achieving these ends requires a process by which communities are engaged to participate in problem identification, priority setting, programme design, implementation, monitoring and evaluation (Table 3). A study in Zaire investigated the effect of community participation in planning and implementation of malaria treatment delivery compared to standard treatment delivery through health centres [49]. Significant reductions in the mean malaria incidence occurred when communities were actively involved in planning and implementation compared to being passive recipients of treatment through health centres [49]. Similarly a study in Cuba, found that community participation that included self-identification of problems and locally derived solutions resulted in sustainable behaviour change and significant reductions environmental risk factors for dengue fever [61].

**Table 3:** Components of the process by which communities are engaged to participate in communicable disease control and elimination programmes and the references in which these components occurred.

Process	References
Engagement of key community stakeholders	[31,33,36,49,50,55-58,60-62,65,67,70,74,78,83,85,86,88,90-94,98-100,104,106,107,110,112]

Promotion of partnership approach	[31,33,36,55,58,60,61,65,70,74,78,85,86,90-94,106,107,110]

Community participation in problem identification and priority setting	[31,33,58,60,61,70,85-87,92,93]

Community participation in programme design	[31,36,55,58,60,61,85-87,90,91,93,94,112]

Community participation in programme implementation	[14,31,33,36,49,50,55-62,64,65,67,70,74,78,83,85-88,90-94,98-100,104,106,107,112]

Community participation in monitoring and evaluation	[31,57,61,93]

Despite often good intentions, community participation programmes included in this review were often limited to stakeholder engagement, promotion of a partnership approach and implementation of externally determined programme activities. However, the analysis suggests that effective community participation for disease control and elimination can be achieved without engaging communities in all components of the process; and that even when this level of engagement is achieved, it does not necessarily equate to inclusive participation. Rather, community participation can be additionally influenced by factors including community characteristics, health and disease priorities, anatomy of political system, integration of programme into primary healthcare goals, level of decentralization of resources, intersectoral collaboration, incentive systems and administrative structures of the programme (such as adequate financial and human resources, training and supervision) which are addressed elsewhere in this review. Nonetheless, a programme of community participation lacking many of the process components can result in participation that is narrow in scope, static, non-inclusive of vulnerable populations and ultimately ephemeral and unsustainable [83].

Promotion of the involvement of communities in all components represented in Table 3 suggests a push towards a democratic style engagement in health despite the ethos of community participation being communal action for the greater good. This is further reinforced in the selection of community volunteers where democratic processes were considered important for the optimal functioning, usage and support of newly introduced volunteer systems [31,36,49,59,62,67,91,94,98]. Despite this, many papers included in this review that detailed programmes utilising community volunteers, described their selection as having been made by community leaders or local health workers with varying degrees of success [50,57,83,88,90,99,100,112]. The advantage of this approach was suggested to be the ability to ensure representation of different stakeholder groups (women, ethnic and religious groups) and selection of a community member with the necessary skills to perform the role adequately [64,90,92].

**Figure 16:**
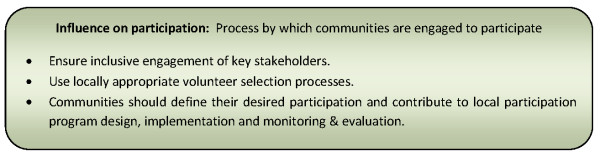
**Summary of considerations for community participation programmes relating to the process by which communities are engaged to participate**.

It is suggested that democratic processes in the selection of community volunteers are not necessary to motivate participation, particularly in countries with a long history of autocratic political rule or a tradition of civil service [99,110], however, designated volunteers may require longer time periods to establish trust in the community [110]. Problems hindering motivation, performance or attrition of community volunteers were usually not related to the process by which they were elected. Rather socio-cultural and gender disparities between volunteers and the communities they serve as well as the level of administrative structure and support of the programme by the formal health system (i.e. lack of financial support, poorly functioning health committees, lack of supervision and expectations underlying volunteerism) affected the success of volunteer systems [31,36,50,83,99,112]. Figure 16 presents a summary of considerations relating to this influencing factor for community participation programmes.

#### Congruence of external targets and local priorities

Community participation can be influenced by how significant the problem is perceived by communities [92]. Health is rarely viewed in isolation to other aspects of daily life and a lack in understanding of disease impact can diminish its priority status [74,84,86,99,101,102,105,108,112]. Selective disease control programmes may, therefore, be perceived as extraneous and lack support. Similarly, a consequence of disease suppression following successful elimination campaigns is waning enthusiasm for participation in prevention and surveillance activities [68,77,108]. In contrast, where disease epidemiology and popularization of campaigns propel it into public interest, participation in intervention measures can be rallied around a shared motivation to conquer the burden of disease in their community [61,63,67,68,77,108].

**Figure 17:**
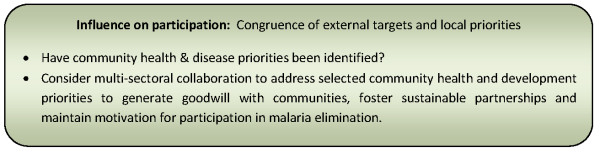
**Summary of considerations for community participation programmes relating to the congruence of external targets and local priorities**.

Contemporaneous attendance to community priorities such as construction or improvement of roads, housing, public buildings, water and sanitation systems and agricultural modernization in conjunction with disease prevention measures have been an important component of some programmes [57,59,86]. Such activities that address issues of health, development and economic significance in communities; provide immediate, tangible benefits; act as an anchor for participation in externally derived programmes; foster confidence in the reciprocity of community and health authority benefits; and sets the tone for future sustainable partnership approaches to disease control [61,67,70,71,74,80,85,88,91,106]. However, a note of caution suggests that proposals for attending to community priorities need to remain within the scope of capabilities of disease control programmes or within the possibilities that may be achieved with intersectoral collaboration [93]. In addition, with socioeconomic reforms creating income disparity and inequalities, it is manifest that community priorities can be dynamic and diverse across socioeconomic strata [65,107]. It is hence not feasible for health authorities with limited resources to build motivation for participation in national disease control or elimination programmes solely on attendance to each community's development needs [93]. Figure 17 presents a summary of considerations relating to this influencing factor for community participation programmes.

### Government and civil society level determinants of participation

#### Political environment

Community participation programmes need to consider in their design, the political environment in which they are operating. Historically, whether motivated by a genuine spirit of collectivism or coerced by a fear of higher authority, community participation for disease control and elimination seems to have been most successful during periods of strong socialist rule in countries such as China, Cuba and Nicaragua [102,107,113]. In such political environments, community participation is aided by policy-making that is directed towards social welfare and development, economic stability, prevention of extreme inequality, a focus on civil service and endorsement of primary health care principles [107,111,113]. Politicization of disease control interventions has also been used as a tool to mobilize communities for mass action in South America [113]. In authoritarian or colonial regimes, human, technical and financial resources are less of a constraint on disease control programmes allowing a focus on intersectoral coordination and mass mobilization of human and material resources [14,73,97]. Therefore, community participation programmes for disease control and elimination operating in these contexts benefit from the efficiency of centralized planning, decentralized implementation, large scale, rapid mobilization of resources and improved access to services [107,111,113]. Such approaches to community participation for disease control or elimination are not viable under democratic governments, with capitalist economies and individualistic motivations [73,113].

**Figure 18:**
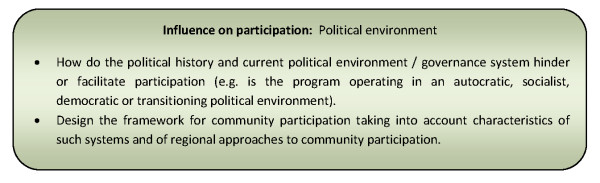
**Summary of considerations for community participation programmes relating to political environment**.

In addition to an understanding of the influence of political philosophies on participation, designing community participation programmes requires consideration of the effect of transitioning political environments. For example, in Melanesian countries, systems of governance are transitioning from a traditional tribal structure to one of post colonial Western democracy, resulting in considerable disparities in health between urban and rural areas [55]. Community participation programmes attempting to address these disparities will need to consider potential variances in social mechanisms that may arise in such countries. In a further example, dramatic political changes in fewer than two decades in Cambodia; from the violent autocracy of the Khmer Rouge to civil war then to multi-party democracy in 1993, has resulted in changing ideas about the role of government in health and development [102]. The breakdown of collectivism, 'community spirit, shared identity and trust,' and a move towards democratic processes and individualism, has brought with it a decline in mass participation for communal benefit and an abdication of community responsibility for health promotion and prevention activities [102,110]. Community participation programmes have therefore needed to remain dynamic and responsive [55,107]. Figure 18 presents a summary of considerations relating to this influencing factor for community participation programmes.

#### Government advocacy and support

Government advocacy and support is suggested as imperative for legitimising disease control and elimination programmes and motivating mass input as well as providing institutional roots from which to sustain community participation. In countries that have experienced the collapse of socialist regimes, transitioning governance systems, violent autocracies, civil war and extreme inequalities arising from economic policies, governments will need to demonstrate leadership, benevolence and commitment to re-establishing trust and a spirit of social unity [88,102,104,113]. In addition, with the growing view of health and development being the duty of governments, building cooperation and partnerships between governments and communities and supporting this with clear national policies that elucidate roles and responsibilities of each, is necessary for creating a solid foundation for community participation [88,102,104,105].

Government advocacy, mandates for national health care delivery and their supporting mechanisms have had a substantial influence on the success of participation at the grassroots [33,64,65,67,81,84,88,90,107,113]. Governments also have the influence to stimulate social change with an impetus toward cooperative working and volunteerism necessary for disease control and elimination programmes [50,99]. For example, the eradication of schistosomiasis in Guangxi Province, China in 1992 was the result of 40 years of government commitment to community participation, upheld by centrally developed policies guiding multi-sectoral collaboration, operational research, encouragement and reward for initiative demonstrated at the local level and a focus on prevention [107]. In addition, the Maoist approach of integrating public health action into the national cultural identity was an important strategy for successfully motivating community participation for disease prevention [107].

Policy decisions to guide allocation of funds, intersectoral collaboration, cost recovery and incentive mechanisms, training and supervision and dissemination of information must be addressed at the highest level by stable governments to provide legitimacy for implementers of the community participation programme [56,77,79,84]. Governments are also able to provide a central system for coordination, technical support, monitoring, evaluation and rapid response [56,113]. Strong programme administration from governments is recognized as importantly providing community participation programmes with the infrastructure for comprehensive, reliable and integrated health care delivery systems; coordinated intersectoral collaboration at all levels; efficient allocation of resources; the impetus to project activities at the periphery; and the ability to overcome restraints [50,58,62,64,67,80,92,94,104]. In developing countries without the resource capacity to provide such infrastructure, governments have been able to leverage their commitment to disease control and elimination to obtain additional donor funding [80,98].

**Figure 19:**
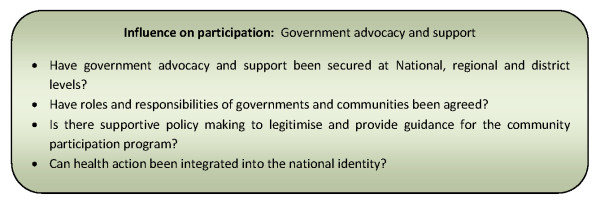
**Summary of considerations for community participation programmes relating to government advocacy and support**.

Large community participation programmes operating outside government support may lack centralized administrative linkages as well as human and financial resources to sustain them [31,93]. A common theme of the papers included in this review was the understanding that to achieve sustainability of a national disease control or elimination programme, government support is required on an enduring basis. Governments and donors essentially provide the machinery through which financial, technical, operational and institutional support can be delivered in order to capitalize on and sustain the benefits of community participation for effective disease control and elimination [14,57,75,84,93]. Figure 19 presents a summary of considerations relating to this influencing factor for community participation programmes.

#### Decentralization of power and resources and use of community assets

It is suggested that decentralization of decision-making to the local level can reduce human resistance to interventions and improve community participation in disease control and elimination programmes [33,60,61,67,86,91,115]. However, the importance of central planning and decision-making for interventions based on epidemiological parameters suggests that it is not viable for national programmes to devolve this control to the community level [14,56,111]. Participation without community-level decision-making regarding intervention strategies can still be enormously effective particularly for disease elimination programmes, however, marked centralization of programmes may leave communities with a sense of powerlessness that is deleterious to community participation [14,68,79,81,93]. Therefore, centralized design with decentralized implementation that harnesses grass roots knowledge and relies on locally derived strategies for maximising community participation is suggested as being the most feasible and successful approach for national disease control and elimination programmes [61,103,107].

The primary advantage of programme decentralization is that it is able to capitalize on community assets to increase accessibility and acceptability of interventions and promotes participation in their implementation [36,49,55,57,62,64,65,68,69,71,79-81,91,92,98,106,116]. It is also suggested to address the constraint of personnel shortages in resource poor countries and harness the influence of youth as change agents [51,59,70,71,73,76,112,116]. However, it will be important to prevent the perception that decentralization is a process for outsourcing implementation or devolving the responsibility of disease control to communities [59]. Ideally, decentralization is suggested be a mechanism that includes liaison between National, Provincial and District level coordinators to negotiate the implementation of more distally derived solutions and mobilization of adequate resources to facilitate programme implementation at the local level [33,56,57,62-64,70,76,85,90-92,98,111].

**Figure 20:**
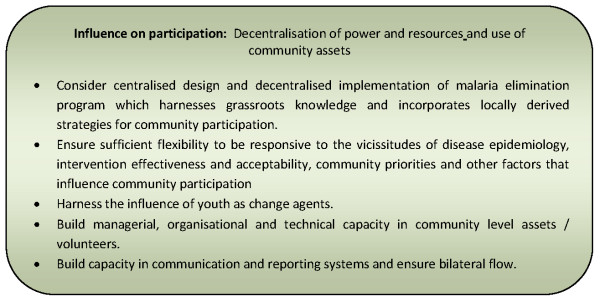
**Summary of considerations for community participation programmes relating to decentralisation of power and resources and use of community assets**.

A number of challenges to decentralization have been identified in the papers reviewed. Firstly, a history of vertical disease control programmes may have contributed to communities considering the implementation of disease control measures as the responsibility of health authorities and therefore not provide their full cooperation in attempts to decentralize such activities [33,74,83,89,103]. Secondly, despite the institution of user fees advocated by some as a mechanism for financial empowerment of health services at the community level, they may also intimate that participation in disease control programmes is optional and that the programme lacks national commitment [56,113]. Thirdly, despite the provision of adequate material and human resources, poor capacity at the periphery with regards to managerial, organizational and technical skills can result in irregular programme implementation and interrupt the momentum of community participation [58,63,93,104,105,109]. Finally, inadequate communication, referral and reporting systems implemented to support decentralization can create uncertainty with regards to roles, responsibilities and progress [31,84]. Figure 20 presents a summary of considerations relating to this influencing factor for community participation programmes.

#### Health authority commitment to Primary Health Care (PHC)

Constraints in financial resources, human resistance to programmes and the lack of adequate public health infrastructure, particularly in remote regions, were fundamental reasons for failures of vertical health projects and the motive for a shift to community-oriented primary health care (PHC) systems in many countries [57,62,67,92,103]. The PHC approach calls for a broad-based establishment of fundamental health services rather than disease-specific interventions and hence selective disease control and elimination programmes operate counter to PHC principles [65,82,91]. With community participation being intimately linked with PHC, it is suggested that controlling or eliminating single diseases may be facilitated if integrated with local-level health and disease priorities and contributing to a strengthening of the PHC infrastructure [49,57,59,64,65,67,76,80,83,84,91,92,94,98,100,105,111-113]. This will be discussed further in the following section.

**Figure 21:**
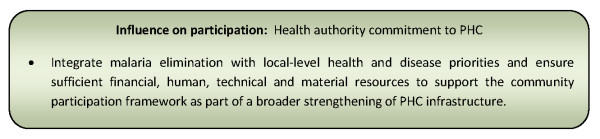
**Summary of considerations for community participation programmes relating to health authority commitment to primary health care**.

At the forefront of PHC systems were established and trusted community level health workers who provided health information, preventative, diagnostic, curative and rehabilitative services. With adequate resources, training and supervision, these pre-existing, imbedded networks of health care providers are best placed to motivate community participation around selected interventions [57,67,101,110-112]. Figure 21 presents a summary of considerations relating to this influencing factor for community participation programmes.

#### Multisectoral collaboration and integration of programme into broader development goals

##### Multisectoral collaboration

Multisectoral collaboration has been an important contributor to the success of disease control and elimination, in part through its motivating influence on community participation [14,33,58-64,69-71,76,85,92,97,105,107,111]. Collaboration has ranged from peripheral contribution of multiple sectors to community derived solutions for risk mitigation, through to multi-level, multisectoral collaboration that is centrally legislated and coordinated. Sectors engaged in communicable disease control and elimination campaigns in the past have included Departments of Forestry, Agriculture, Water, Sanitation, Education, Information, Propaganda, Communication, Public Works, Law Enforcement and Commerce as well as religious development schemes, Women's Federations, Youth Leagues, private mining and chemical industries, NGOs and other civic groups [62,70,97,107,111]. An important element of the global smallpox eradication campaign and other disease control and elimination programmes has been collaboration with research institutions. Epidemiological, anthropological and sociological research has been vital to identifying barriers and facilitators of community participation and guiding implementation of effective interventions based on sound evidence [33,59,66,68,82,107]. With the interconnection between animal and human health it has also been suggested that avenues for linkage with the animal husbandry sector should be explored also [57].

A feature of Taiwan's malaria elimination experience was the active involvement of the military in all phases of the campaign, but they were of particular importance for supporting communities in disease surveillance activities and rapid response (including resource mobilization and disease containment) during malaria outbreaks in the post-elimination phase of the programme [14]. Other advocated advantages of multisectoral collaboration that promote community participation were the creation of social and organizational linkages, utilization of influential human resources (such as teachers and sector leadership), and the provision of technical and material resource support for community level problem solving and preventative actions [33,58,60-63,65,69,86,92,105]. Sustainability of a community participation programme may also be enhanced by multisectoral collaboration that generates economic incentives [71,76]. In the schistosomiasis elimination campaign in Guangxi Province, China, collaboration with the agricultural sector from the programme's outset sustained community participation by ensuring that control measures also boosted agricultural production [76].

In contrast, a lack of collaboration can result in intersectoral competition for the limited time and capacities of communities to participate in development programmes [83]. In addition, multisectoral collaboration and policy development that occurs only at the periphery can lack national-level recognition making expansion of programmes difficult [85]. The inclusion of government departments and public and private organizations at all levels (central, regional, district and local) at the outset in planning, implementation, research and evaluation is therefore suggested as important for national communicable disease control and elimination programmes [64,106,107,111]. However, the post-war development of powerful insecticides that allowed immediate and visible reductions in vector-borne disease transmission is suggested to have resulted in the breakdown of intersectoral operations and lead to isolated and parallel workings of the otherwise complementary sectors of agriculture, transport, energy, urban planning, engineering, sanitation and public health [73]. Therefore, although linkages between these sectors are advocated, due to weak government systems, many developing countries lack the capacity to form multisectoral collaborations for sustainable development activities that support communicable disease control [73,104].

##### Integration into broader development goals

A number of papers proposed the integration of selective disease control into broader development goals as a means of promoting community participation. Integration is suggested as being of particular benefit to community participation when selective disease control efforts produces less perceptible community benefits than development activities such as sanitation, provision of safe water, income generation schemes and housing improvements [31,59,74,103,104]. For example, community participation in vector control for Chagas disease was enhanced by its integration with housing improvement projects in Brazil, Argentina and Bolivia [59,104]. Conversely, community development may be consolidated by progress made towards selective disease control [105]. In addition, selective disease control or elimination programmes with well functioning community surveillance systems may provide a valid entry point to surveillance of other diseases of public health and economic significance [106].

Integration of selective disease control programmes with PHC is also suggested to enhance the quality of community participation [92]. Integration at the national, district and community levels may strengthen the PHC system, better address the perceived priorities of the local population, increase utilization of health centres and allow resource efficiency and utilization of existing community assets [59,67,84,94,98]. Use of existing community health workers with polyvalent functions capitalizes on the rapport, leadership, access and acceptability they may have established in their community [93,112]. In addition, by packaging PHC interventions or forming linkages with other diseases such as HIV, TB, malaria and polio, neglected diseases can benefit from shared intervention objectives and the financial support, coverage and community commitment achieved by high profile diseases [91,98,109]. Further, the enthusiasm and momentum generated by disease elimination programmes has also provided a valuable entry point to other PHC interventions such as extension of immunization programmes, oral rehydration, and family planning in remote areas [80]

**Figure 22:**
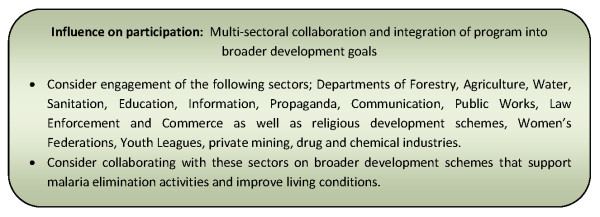
**Summary of considerations for community participation programmes relating to multi-sectoral collaboration and integration of programme into broader development goals**.

A study investigating the effect of intersectoral collaboration on preventative actions for dengue control in Havana, Cuba found that strengthening intersectoral coordination significantly improved community participation. In conjunction with an empowerment approach, intersectoral collaboration had an even greater impact on participation than intersectoral collaboration alone [89]. Despite the potential motivating effect on community participation, integration of selective disease control and elimination programmes into broader development goals or PHC systems do not always result in progress towards reductions in disease transmission [31,57]. Dilution of focus on the targeted disease and the magnitude of PHC needs in a community may overwhelm community health workers and volunteers, thereby suggesting that participation is best improved if directed toward well-defined activities that bring tangible results [31]. In addition, integration for elimination may be challenging when the targeted disease is not perceived as a community priority or as life threatening and is in conflict with resources for more pressing issues such as child survival or deadly epidemic diseases [80]. Figure 22 presents a summary of considerations relating to these influencing factors for community participation programmes.

#### Financial and human resources

##### Human resources

Adequate human resources that have been trained, supervised and ideally institutionalized are vital to the foundation of community participation for disease control and elimination programmes [31,33,36,50,56,57,59,69,84,98,102,104,106,111,112]. This was particular important for successful disease elimination in Cyprus, Cameroon, India and China where an enabling environment for elimination was created by diligent quality control, cyclical training and regular supervision [68,76,81,82,97]. In addition, a key element of sustainable community participation is suggested to be an organizational and administrative framework that provides a mechanism through which communities establish capacity, generate resources, identify roles and responsibilities, outline expectations and create systems of accountability [31,57,90,91,94,105].

However, adequate human resources, training and supervision can be limited by insufficient investment in community participation by programmes and by competing economic interests that create labour shortages such as seasonal employment on commercial farms [88,98]. In addition, the skills required by a community volunteer to carry out the duties required (communication in multiple dialects, literacy and basic arithmetic functions) may restrict the availability of human resources in the community [100]. Community members with these proficiencies (such as health staff and teachers) may not have the capacity for project management, training, supervision or implementation of activities in addition to their standard workload [49]

##### Training

The acceptability of human resources also affects community participation. The nature of duties carried out by community volunteers and the quality of their training may have a significant impact on their ability to mobilize community action. A community volunteer that provides health promotion rather than curative services may have insufficient status to motivate participation [99]. Volunteers may become dissatisfied by the limited nature of their role in selective disease control programmes [49]. In addition, a rapidly trained community volunteer may lack legitimacy in the eyes of the community [99,112]. This can be further exacerbated by a insufficient supervision by health staff [99]. A study in Zaire, where problems relating to volunteer malaria officers were observed, qualitatively described that these workers were eager to receive training that broadens the scope of their role and that they desired formal recognition in the health system hierarchy so as to allow career progression [49].

Volunteer training packages often consisted of selective disease information, preventive, diagnostic and treatment methods consistent with a medical model approach which may be at odds with more holistic community views of health and disease management [83,99]. Therefore, it is suggested that training be relevant to local conditions and incorporate a broader range of skills [84,111]. Training packages ranged from several days to several months, however many programmes recognized the importance of refresher training and continuing education programmes to maintain skill levels and motivation [63,76,81,83,106,109]. Some programmes packages have included training in locally relevant PHC conditions, concepts in village self-reliance and techniques for mobilizing community action, participatory and empowerment processes, and management of accounts and budgets for communities instigating income generation schemes [33,56,83,84]. A comprehensive, two-month training programme of itinerant health workers in Cameroon included principles of health education, community organisation, school health, environmental health techniques and principles of disease control that were tailored to local conditions [93].

Though often easily established, village leadership committees used to coordinate the implementation of health and development programmes at the community level may be weak or non-functional due to inadequate management training [31,88]. Therefore, training has also been suggested in topics such as cooperative teamwork, communication skills, generating and adhering to guidelines for committee member interaction and role fulfilment as well as in project management and technical skills to equip them for implementation activities [31,33]. Central level training packages to facilitate health management staff in generating and sustaining community participation should also include the development of skills in situational analysis, institutional capacity assessment, strategic planning, technical issues, community engagement and competency in negotiation [58,70]. Importantly, it has also been suggested that at all levels, training needs should be pre-assessed and packages tailored to the outcomes of such appraisals [61,70].

Genuine commitment to building sufficient capacity for training and human resources in developing countries was demonstrated in Cameroon in the 1990s, where predominately donor funding and technical assistance from a tertiary institution established a parasitic diseases research centre over a seven year period. This research centre carried out community-based studies of schistosomiasis and other parasitic diseases which has been instrumental in directing control strategies and facilitating integration of programmes into the PHC system [67].

##### Supervision

**Figure 23:**
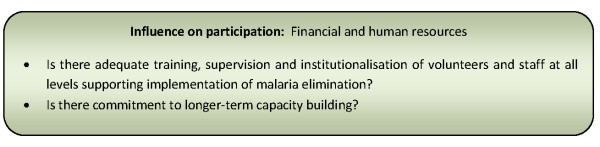
**Summary of considerations for community participation programmes relating to financial and human resources**.

Regular supervision of community volunteers and health workers has been an important element of many programmes and provides an avenue for consolidation of skills, expression of concerns and provision of epidemiologic feedback to maintain motivation [58,63,69,83,84,93,94,99,106,113,117]. Patronage by respected health workers has also had the effect of increasing volunteer credibility and acceptability in the community [99]. The vital role of regular supervision is highlighted by examples of the consequences of its absent or inadequate provision [84,93]. Lack of supervision and support, often compounded by weak health infrastructures, result in large numbers of community volunteers and health workers losing motivation, activity levels decline and attrition rates increase considerably [84,93,99,113]. Figure 23 presents a summary of considerations relating to these influencing factors for community participation programmes.

##### Techno-financial support & implementation style of locally embedded development agencies

In many developing countries, inadequate health infrastructure, insufficient decentralisation and lack of resources mean government services only reach a proportion of the population [52]. Civil society represents a valuable source of vital resources, technical and training capacity and has links to poor and isolated communities that lack access to government health services [52,58,65,80,81,85,99,100]. Of additional benefit to community participation programmes is civil society's culture of volunteerism, the mass appeal of some Non-Government Organizations (NGOs) and their extensive histories in stimulating and sustaining large scale volunteer movements [57,99]. Imbedded NGOs can also have an intimate understanding of local conditions and are able to be more flexible and responsive to community needs [57,99].

Some potential challenges in utilizing NGOs for community participation in disease control and elimination are that they may have high turnover of competent staff, which can effect programme continuity and there may be difficulties in navigating the divide when disease control programme staff or activities are in conflict with the philosophies of the locally imbedded NGO [57]. The implementation style of development agencies, NGOs and other civil society organisations are also considered to influence whether communities participate actively or passively [57,102].

**Figure 24:**
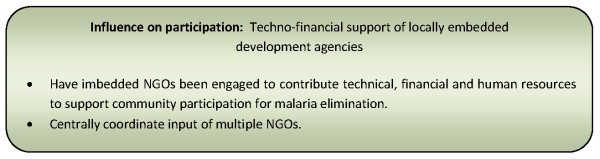
**Summary of considerations for community participation programmes relating to techno-financial support of locally embedded development agencies**.

Nonetheless, as well as their ability to mobilize communities, NGOs may have the influence to rally other organisations and private industry to a cause generating further financial, technical, material and human resources to support community participation in disease control and elimination [71,80,85]. Embedded NGOs with effective relationships with governments and health authorities are also in a position to effectively lobby for the promotion of active community

participation in order to achieve desired public health targets in relatively short timeframes [118]. Figure 24 presents a summary of considerations relating to this influencing factor for community participation programmes.

## Discussion

The findings of this atypical systematic review with qualitative synthesis of published literature over the last 60 years, has elucidated the complex architecture of community participation for communicable disease control and elimination and provided guidance for planning community participation programmes for malaria elimination. Some limitations, however, do exist. The lack of coherent quantitative studies to support findings of the content analysis is a limitation of this systematic review. It is also possible that not all relevant papers were captured due to the search strategy being restricted to English language papers and published material. Grey literature may contain a number of examples of community participation but were not included in the review due to the lack of comprehensive access to this data source. These limitations may have restricted an exploration of regional patterns in influences on community participation. It is also recognized that the qualitative method used for this analysis is vulnerable the introduction of bias. To minimize this risk a transparent reporting of method and an inductive approach to data synthesis were used which included self and peer reflection processes [33].

The conclusions of this review are consistent with a review carried out in the 1980s which found that there can be no universal model for effective community participation [119]. However, this systematic review goes further and identifies multi-level, interacting influences on participation and proposes corresponding considerations for the design of participation programmes to support malaria elimination. This review also suggests that although no single model for community participation is possible, regional models may be possible based on similarities in governance systems or approaches to community participation between countries of the same region. This concept, however, requires further exploration. Considerations of paradigms, definitions and approaches to community participation have provided useful guidance for the design and implementation of participation programmes; however, failures continue to occur as a result of lack of understanding of the breadth of factors that influence participation that have been highlighted by this review, as well as insufficient allocation of funding to build adequate long-term infrastructure for community participation [22,38,39,99].

### Where is the evidence to attract investment in community participation?

Although investment in community participation is usually based on the assumption that such efforts will contribute to reductions of disease transmission; this systematic review has revealed a deficiency in robust evidence to support this claim which may be responsible for the lack of prominence community participation is assigned in programme budgets. Of further concern is the message purported by inadequate community participation budgets; that the use of community assets and the promotion of a community ownership and self-reliance approach will substitute for more comprehensive investment required for large scale community-based activities to support disease elimination programmes.

After 60 years of research and comprehensive discussion regarding the merits of community participation for disease control and elimination, there has been a failure to produce sufficient rigorous evidence of its effectiveness in reducing disease transmission. With examples of effective top-down approaches to disease elimination, and a lack of evidence of the significant public health benefit of community participation, it is difficult to lobby donors and policy makers to make significant long-term investments in the infrastructure required to support the 'people' component of health systems [39,120]. Much has been written on the human behavioural factors that influence disease transmission and this paper contributes to the literature on issues that influence community participation. Is it not now time to harness this vast resource, design locally appropriate, inclusive and responsive community participation programmes and carry out empirical research with sufficient epidemiological astuteness to contribute to an evidence base from which to leverage adequate future investment?

Quantitative research investigating the effectiveness of community participation in reducing disease transmission has all but been discounted in the literature. It has been criticized as being too difficult and it is often too complex to differentiate out the confounding effects of variations in social and ecological conditions, intervention strategies, differences in local resource availability and quality of existing health infrastructure [21,120]. Due to these perceptions, focus has shifted from evaluating community participation using biological indicators to using behavioural or process indicators and measures of social impact [33,66,120,121]. While these indicators will provide valuable quality monitoring for community participation programmes, they do not contribute to the empirical evidence required to stimulate significant investment from international donors and in-country policy makers. Until it can prove itself an effective public health intervention, genuine community participation is at risk of remaining on the sidelines of public health policy.

### Implications for malaria elimination

Countries that were successful in the malaria eradication campaign of the 1950s primarily had strong health systems and advanced malaria control programmes [2]. In recognition of this, the current global malaria elimination campaign calls for a health systems strengthening approach to provide an enabling environment for programmes in developing countries [122]. However, this may be more challenging than anticipated. Globalization and macro-economic reforms that promote free market economies have eroded social cohesion that underpins community spirit and participation. In addition, ensuing health inequalities and an abdication of community responsibility for health care delivery have placed health systems under further pressure to respond to meet service requirements consistent with people's increasing expectations and varying priorities [102,110,123]. The capacity of health systems' in developing countries to effectively respond to these challenges as well as support the demands of competing selective disease elimination programmes can especially limited [123].

Thirty years after the Alma Ata declaration, international public health thinking has once again returned to a PHC focus to systematically address health inequalities and build more sustainable mechanisms for the delivery of universal health care [123]. A fundamental feature of the renewed push for a comprehensive PHC approach is the accompanying agenda of reforms it includes to better gear health systems towards support of the PHC approach, scale-efficient systems thinking and increasing multi-sectoral involvement [123,124]. It is now understood that, '*every intervention, from the simplest to the most complex, has an effect on the overall system*' [124]. Therefore, despite the well-meaning intentions of the health systems strengthening approach to malaria elimination, continued push for selective disease elimination programmes accompanied with the considerable global enthusiasm and political and financial support they rally, may once again place pressure on health systems to divert from their comprehensive PHC objectives.

Another important challenge of strengthening health systems for malaria elimination is establishing the widespread understanding that people are an intrinsic component of health systems, not just as recipients of health care but as drivers of the system itself [124,125]. Past failures to invest adequately in research and infrastructure to create sustainable community participation frameworks is evidence of a lack of understanding of the importance of people in health systems effectiveness. Creation of an enabling environment for malaria elimination should necessarily include the fostering of 'competent communities.' This concept arises from the HIV/AIDs literature which highlights the importance of greater attention to 'community readiness' for programme implementation, where potential obstacles are addressed and social assets mobilised in preparation for optimal uptake of interventions and utilisation of health services [126-128]. Health system effectiveness may be significantly enhanced when communities are primed to derive maximum benefit from programme implementation. This groundwork is often neglected in the push to scale up interventions and meet short-term targets set by external funding agencies.

The scale of community participation required for malaria elimination cannot be achieved with an ad hoc approach to its design and implementation. Rather, large-scale cooperative, collective and sustained action requires an appropriate organizational framework for coordination, credibility and accountability. In the past, health planners have used community participation strategies as a means of navigating programme constraints such as scarce financial and human resources and 'human resistance' to interventions [92]. Community participation for malaria elimination requires significant investment in people as well as the structure and capacity to support this investment, making the design of an appropriate framework at the outset an important first step [24]. This review emphasizes the importance of avoiding the creation of additional external structures, local-level participation can be incorporated into existing social, organisational and institutional structures through which health authorities decentralize and support implementation of elimination measures, coordinate multi-sectoral collaboration, exchange epidemiological progress and feedback with communities as well as providing technical and resource support [33,64].

Above all, integration of a community participation framework for malaria elimination into the broader PHC strategy will be vital. It will have important and reciprocal benefits of strengthening and maintaining health systems on a course towards achieving health equity, while benefiting from established scale-efficiency and harnessing the ensuing re-engagement and inclusive participation of communities in health care delivery for more effective and sustainable malaria elimination. Despite the application of this analysis to the context of the current global malaria elimination efforts, the results of this systematic review of community participation in communicable disease control and elimination could and should be extended to other diseases of poverty such as HIV/AIDS, TB and neglected diseases. This may harmonize efforts at building competent communities for communicable disease control and optimise health system effectiveness.

This review has comprehensively elucidated the multi-level factors that influence community participation for communicable disease control and elimination, and in doing so, contributes to the understanding of the 'people' component of health systems, an outstanding priority identified by the malERA Consultative Group on Health Systems and Operational Research for malaria eradication [125].

## Conclusions

Constraints in financial resources, human resistance to programmes and the lack of adequate public health infrastructure, particularly in remote regions, were fundamental reasons for failures of vertical health projects and the motive for a shift to community-oriented PHC systems in many countries [57,62,67,92,103]. The cornerstone of PHC is community participation, which has played a critical role in successful disease control and elimination campaigns in many countries. Despite this, its benefits for malaria control and elimination are yet to be fully realized. This may be due to a poor understanding of the constructs of participation in developing countries as well as inadequate investment in the 'people' component of health systems including essential infrastructure and resources to support the scale of and coordination of community participation required for malaria elimination. The findings of this review of 60 years of published literature on communicable disease control and elimination draws attention to a deficiency in the evidence base for the effectiveness of community participation from which to lobby for significant long-term investment. In addition, the complexity of multi-level, interacting influences on participation identified in this review, attests to the inability to create a global model for community participation within health systems, however, it emphasizes the importance in community participation having a position in every system. Despite the challenges, community participation remains an essential component of any attempt to eliminate malaria; a disease that unlike small pox, currently has no vaccine, is not easily recognizable without appropriate diagnostic tools and can have latent or persistent human infection[129]. It is recommended that the application of the results of this systematic review be considered for other diseases of poverty in order to harmonize efforts at building competent communities for communicable disease control and optimise health system effectiveness.

## Authors' contributions

Planning for systematic review carried out by JA, MT & AV. All authors contributed to the design of the original coding matrix. The systematic review, data analysis and manuscript drafting was carried by JA with support and contributions from all authors. All authors have read and approved the final manuscript.

## Conflicts of interests statement

The authors declare that they have no competing interests.

## Supplementary Material

Additional file 1 **Summary of papers meeting selection criteria and included in the analysis**. Click here for file

Additional file 2 **The matrix used to analyse influences on community participation**. Click here for file

Additional file 3 **Summary of the quantitative evidence: study characteristics and selected results**. Click here for file

Additional file 4 **Summary of lessons learned from quantitative research papers**. Click here for file
