# Supplementary material for: The architecture and effect of participation: a systematic review of community participation for communicable disease control and elimination. Implications for malaria elimination
Source: Malar J. 2011 Aug 4;10:225. doi: 10.1186/1475-2875-10-225 (PMC3171376; doi:10.1186/1475-2875-10-225)
Supplement: Additional file 1 — Summary of papers meeting selection criteria and included in the analysis. [file 1475-2875-10-225-S1.PDF]

Additional file 1: SUMMARY OF PAPERS REVIEWED

**Observational:**	**SUMMARY OF ALL PAPERS**			
Descriptive ***(Case reports / Case Series)***	**Author / Year**	**Communicable diseases addressed**	**Country**	**Research Method Used** (qualitative, quantitative or mixed methods)
	(Garfield and Vermund 1986)	Malaria control	Nicaragua	Case report (1980s) (plus M&E results)
	(Garfield 1999)	Malaria control	Nicaragua	Case report (1990s) (plus M&E results)
	(Chen 1991) and (Yip 2000)	Malaria elimination	Taiwan	Case reports
	(Kaneko 2010)	Malaria elimination	Aneityum, Vanuatu	Case report
	(Mantra 1992)	Malaria control	Indonesia	Case report
	(Sleigh, Xueming et al. 1998)	Schistosomiasis	Guangxi Province, China	Case report (plus M&E results)
	(Azhar, Lubis et al. 2010)	Avian Influenza control	Indonesia	Case report
	(Ruebush and Godoy 1992) I & (Ruebush, Zeissig et al. 1992) II & (Ruebush, Zeissig et al. 1994) III	Malaria control	Guatemala	Case report (plus M&E results)
	(Harkins, Drasbek et al. 2008)	Integrated Management of Childhood Ilnesses (IMCI)	Chao, Peru & San Luis, Honduras (South and Central America)	Case reports (plus M&E results)
	(Fitzpatrick and Ako 2007)	Malaria control	Papua New Guinea	Case report
	(Rojas, Botero et al. 2001)	Malaria control	Pacific Coast of Colombia	Case report (plus M&E results)
	(Nathan, Lloyd et al. 2004)	Dengue control	Caribbean	Case report
	(Malecela, Mwingira et al. 2009)	Lymphatic filariasis	Tanzania	Case report
	(Rajagopalan and Panicker 1984)	Malaria control	Pondicherry, India	Case report
	(Sharma 1987)	Malaria control	Gujarat, India	Case report
	(Hoy, Southavilay et al. 2008)	HIV control	Lao PDR	Case report (plus M&E results)
	(Obregon, Chitnis et al. 2009)	Polio elimination	India and Pakastan	Case report (based on review of primary and secondary data)
	(Chandiwana, Taylor et al. 1991)	Schistosomiasis control	Zimbabwe	Case report (plus M&E results)
	(Kaseje and Sempebwa 1989)	Malaria control	Saradidi project, Kenya	Case report
	(Magnussen, Ndawi et al. 2001)	Schistosomiasis control	Tanzania	Case report (plus M&E results)
	(Ghebreyesus, Alemayehu et al. 1996)	Malaria control	Tigray Region, Ethiopia	Case report (plus M&E results)

	(Lin-hua, Hui-lin et al. 1991)	Malaria control	China	Case report
	(Okanurak and Sornmani 1992)	Malaria control	Thailand	Case reports of 2 programs
	(Perez, Lefevre et al. 2007)	Dengue control	Havana, Cuba	Case study (Historical)
	(Richards, Gonzales-Peralta et al. 1996)	Onchocerciasis control	Nigeria	Case report
	(Guthmann, Calmet et al. 1997)	Leishmaniasis control	Peru	Case report
	(Constantinou 1998)	Malaria elimination	Cyprus	Case report
	(Edungbola, Withers et al. 1992)	Guinea worm elimination	Nigeria	Case report
	(Foster 1978) and (Fenner 1982)	Smallpox eradication	Global	Case reports
	(Cline and Hewlett 1996)	Schistosomiasis control	Cameroon	Case report (plus M&E results)
	(Katsivo, Muthami et al. 1993)	Schistosomiasis control	Kenya	Case report (plus M&E results)
	(Isely 1979)	Integrated control program (Intestinal diseases, helminths, malaria)	Cameroon	Case report (plus M&E results)
	(Bryan, Balderrama et al. 1994)	Chagas disease control	Bolivia, Venezuela, Brazil (South America)	Case report
	(Boelee and Laamrani 2004)	Schistosomiasis control	Morocco	Case report (plus M&E results)
	(WHO 1980)	Smallpox eradication	Global	Case report (plus M&E results)
	(Sam-Abbenyi, Dama et al. 1999)	Guinea worm elimination	Cameroon	Case report (plus M&E results)
	(Loue, Lloyd et al. 1996)	HIV control	USA	Case report (plus M&E results)
	(Kassambara, Poudiougo et al. 1986)	Onchocerciasis control	Mali	Case report
	(Walt, Perera et al. 1989)	Malaria control / PHC	Sri Lanka	Case report (plus M&E results)
	(Shiff 1998)	Malaria control	Tanzania	Case report (plus M&E results)
	(Panicker and Dhanda 1992)	Filariasis control	Southern India	Case report
	(Garcia-Zapata and Marsden 1993)	Chagas disease control	Mambai, Goias, Brazil	Case report (plus M&E results)
	(Gurtler, Kitron et al. 2007)	Chagas disease control	Gran Chaco, Argentina	Case report (plus M&E results)
	(Riji 1992)	Malaria control / PHC	Malaysia	Case report (plus M&E results)
	(Silva, Navaratna et al. 1988)	Malaria control	Sri Lanka	Case report (plus M&E results)
	(Mutalemwa, Kisinza et al. 2009)	Onchocerciasis control	Tanzania	Case report (plus M&E results)

SUMMARY OF RESEARCH PAPERS

	Author / Year	Quality assessment of research papers	Sample size	Communicable disease addressed	Country	Research Method Used (qualitative, quantitative or mixed methods)
**Observational Studies:**						
**Analytical** ***(Correlation, prevalence, cohort, or case-control studies)***	(Jacobs and Price 2003)	Moderate	656	PHC	Maung Russay &Kirivong districts, Cambodia	Quantitative
	(Babu, Behera et al. 2006)	Moderate	7226	Lymphatic filariasis	Orissa, India	Quantitative
**Intervention Studies:**						
RCTs	(Okonofua, Coplan et al. 2003)	Moderate	1896 (pre) + 1858 (post)	STDs	Nigeria	Quantitative
Effectiveness Studies ***(preventative field trials)***	(Kironde and Kahirimbanyi 2002)	Strong	769	Tuberculosis control	South Africa	Quantitative
	(Ramaiah, Vijay Kumar et al. 2001)	Strong	3869	Lymphatic filariasis	Tamil Nadu, India	Mixed methods
	(Hii, Chee et al. 1996)	Moderate	72 + 33	Malaria	Sabah, Malaysia	Quantitative
	(Katabarwa, Habomugisha et al. 2010)	Moderate	1388	Onchocerciasis control	Uganda	Quantitative
	(Toledo, Vanlerberghe et al. 2007)	Moderate	230	Dengue control	Cuba	Mixed methods
	(Sanchez, Perez et al. 2009)	Moderate	250 households	Dengue control	Cuba	Mixed methods
	(Delacollette, Van der Stuyft et al. 1996)	Moderate	30,000	Malaria control	Zaire	Quantitative
	(Castro, Tsuruta et al. 2009)	Strong	900	Malaria control	Tanzania	Quantitative
	(CDI Study Group 2010)	Moderate	4520	Onchocerciasis control / integrated PHC	Cameroon, Nigeria & Uganda	Mixed methods
**Qualitative Studies:**						
	(Khun and Manderson 2008)	Moderate	Approx 100	Dengue control	Cambodia	Qualitative (primarily)
	(Ndekha, Hansen et al. 2003)	Moderate	240	Schistosomiasis control	Zimbabwe	Qualitative (primarily)
