# Supplementary material for: The architecture and effect of participation: a systematic review of community participation for communicable disease control and elimination. Implications for malaria elimination
Source: Malar J. 2011 Aug 4;10:225. doi: 10.1186/1475-2875-10-225 (PMC3171376; doi:10.1186/1475-2875-10-225)
Supplement: Additional file 2 — The matrix used to analyse influences on community participation. [file 1475-2875-10-225-S2.PDF]

Additional file 2: THE ARCHITECTURE OF COMMUNITY PARTICIPATION FOR MALARIA AND OTHER COMMUNICABLE DISEASE CONTROL AND ELIMINATION

INFLUENCES ACTING AT EACH LEVEL OF PARTICIPATION	INTERVENTIONS																	
	MALARIA CONTROL							MALARIA ELIMINATION (Phase I - Getting to Zero)								MALARIA ELIMINATION (Phase II - 'Holding the Line')		
	Personal protection (LLINs, repellants)	Indoor Residual Spraying (IRS)	Diagnosis & treatment (PCD & ACD/MBS where applicable)	Chemoprophylaxis (MDA, IPTi)	Surveillance (parasite and/or vector)	Larviciding and source reduction	Community participation in other disease control & health development programs	Long-lasting Insecticide treated bed nets (LLINs)	Indoor Residual Spraying (IRS)	Diagnosis & treatment of presenting cases (PCD)	Active Case Detection (ACD) (& case investigation)	Chemo-prophylaxis (MDA, IPTi)	Surveillance (parasite and/or vector)	Larviciding & source reduction / adult mosquito eradication	Lessons from other disease elimination programs	Surveillance (parasite and/or vector)	Maintain interventions (vector control, personal protection, diagnosis / treatment)	Lessons from other disease elimination maintenance programs
**INDIVIDUAL**																		
Knowledge and perceptions of disease, its causality, prevention & treatment	[1] [2]		[3] [4] [5] [6] [7] [8] [9]	[10] [11]	[5]	[5] [12] [13] [14] [9]	[15] [16] [17] [18] [19] [20] [21] [22] [23] [24] [25] [26] [27] [28] [29] [30] [31] [32] [33] [34] [35] [36] [37] [38] [39] [40] [41]	[42]	[43]			[42]			[44] [45] [46] [47] [48] [49]	[43]	[42]	[48] [47]
Vulnerability versus resilience (self efficacy, empowerment and mitigation of risk)	[1] [7] [2]		[4] [50] [5] [6] [11] [9]		[5]	[5] [12] [9]	[16] [17] [19] [21] [23] [51] [25] [26] [29] [52] [30] [32] [35] [36] [38] [40] [41]											
Stigma							[18] [21] [23] [30] [38]							[53]	[46] [47] [49]			
Acceptability of interventions or program	[1] [7] [2]		[54] III [5] [3]	[11]	[5]	[5] [12] [13]	[15] [16] [18] [19] [55] [56] [20] [21] [57] [24] [51] [27] [28] [29] [30] [35] [36] [37] [58] [38] [40]		[43]			[42]		[53]	[45] [47] [48] [49]			
Incentive	[1] [2]		[54] [4] [50] [5] [6] [11] [7] [8]		[5]	[5] [12] [13] [14]	[15] [17] [18] [19] [55] [56] [57] [23] [24] [51] [25] [26] [27] [28] [31] [32] [33] [35] [36] [37] [58] [40]			[43]				[53]	[44] [46] [47] [48] [49]	[43]	[42]	[48] [47]
**HOUSEHOLD**																		
Gender roles and power relationships	[1] [7]		[54] [5] [6] [11]	[10] [11]	[5]		[19] [56] [21] [51] [35]								[45]			
Consideration of cultural norms & social mechanisms	[1] [2]		[4] [50] [5] [6] [7]		[5]	[12] [13]	[19] [56] [22] [57] [23] [24] [51] [26] [27] [29] [52] [30] [31] [35] [40] [41]								[45] [44] [49]			
Access (encompassing availability, accessibility, affordability & adequacy)	[1] [59] [7] [2]		[3] [54] [4] [50] [5] [6] [11] [59] [8]	[10] [59]		[12] [13] [14]	[16] [17] [18] [19] [21] [57] [23] [24] [51] [27] [28] [29] [30] [33] [34] [35] [36] [37] [38] [58] [39] [40] [41]			[43]					[44] [46] [47] [48] [45] [49]		[42]	
Urban versus rural implementation	[1]		[54]	[10]			[16] [17] [26]											
**COMMUNITY**																		
Community characteristics (i.e. heterogeneous / divisions or socially cohesive)	[1] [59] [7] [2]		[60] [50] [5] [6] [59]	[10] [59]	[5]	[5] [12]	[15] [16] [17] [19] [55] [56] [20] [22] [23] [26] [31] [33] [36]								[44] [49]	[43]		[44]

Disease epidemiology and complexity of intervention.	[1] [7]		[3] [54] II [50]	[10]		[5] [12] [13]	[18] [24] [28] [29] [30] [31] [34] [36] [37] [41]		[43]			[42]		[53]	[44] [46]	[42]		
Processes by which communities are engaged / empowered to participate.	[1] [7] [2]		[3] [54] [4] [50] [5] [6] [11] [7] [8]	[10]	[5]	[5] [12]	[15] [16] [17] [18] [19] [55] [56] [21] [22] [57] [23] [24] [51] [25] [26] [28] [29] [52] [30] [32] [35] [36] [37] [39] [20]				[43]		[43]		[44] [45]	[43] [43]		
Congruence of external targets and local priorities.			[54] [4] [5] [11] [7] [9]	[10]	[5]	[5] [12] [13] [9]	[15] [17] [56] [20] [22] [23] [24] [51] [25] [26] [27] [28] [29] [32] [33] [35] [36] [39] [40]	[42]				[42]			[44] [46] [49]	[42] [43]	[42]	[44]

GOVERNMENT AND CIVIL SOCIETY

Political environment of program	[59]	[59]	[60] [59]	[59]		[14]	[55] [33]		[43]	[43]	[43]		[43]	[53]	[44]	[43] [43]		
Government advocacy & support	[2]		[3] [60] [4] [50] [5] [11]	[10]	[5]	[5]	[16] [17] [18] [19] [56] [21] [22] [23] [24] [51] [26] [33] [36] [37]		[43]	[43]	[43]	[42]			[44] [46] [47] [48]	[43] [43]	[42]	[44]
Decentralisation of power and resources to the local level and identification / use of community assets.	[1] [59] [2]	[59]	[3] [54] [4] [50] [5] [6] [11] [59] [7] [8] [9]	[10] [59]	[5]	[5] [12] [13] [14] [9]	[15] [16] [17] [18] [19] [56] [20] [21] [22] [57] [23] [24] [51] [25] [26] [27] [28] [29] [30] [31] [33] [34] [35] [36] [37] [58] [38] [39] [40] [41]		[43]		[43]	[42]	[42]	[42]	[44] [46] [47] [48] [49]	[43] [42] [43]	[42]	
Health authority commitment to Primary Health Care.	[7]		[3] [60] [54] [4] [5] [11] [59] [8]	[10] [59]		[12] [13]	[16] [17] [18] [56] [20] [22] [57] [24] [51] [27] [28] [33] [35]			[43]		[42]		[53]	[44] [46]	[42] [43]	[42]	[44]
Multisectoral collaboration and / or integration of program in broader development goals.	[59] [7]	[59]	[3] [4] [5] [6] [11] [59] [9]	[10] [59]	[5]	[5] [12] [13] [14] [9]	[15] [17] [18] [20] [21] [22] [23] [24] [51] [25] [26] [27] [28] [29] [52] [30] [31] [34] [35] [37] [39] [40]			[43]				[53]	[44] [46] [48] [49]			
Financial and Human Resources (with adequate training & supervision).	[59] [2]	[59]	[60] [54] [4] [50] [5] [6] [11] [7] [59] [8] [9]	[10] [59]	[5]	[5] [14]	[15] [16] [17] [18] [19] [56] [20] [21] [22] [57] [24] [51] [26] [27] [28] [29] [52] [31] [33] [34] [35] [36] [37] [58] [38] [39] [40] [41]		[43]	[43]	[43]	[42]	[43]	[53]	[44] [46] [47] [48] [49]	[43] [42] [43]	[42]	
Techno-financial support and implementation style of locally embedded development agencies			[4] [5]		[5]	[5]	[17] [56] [21] [57] [23] [33] [58] [40]							[53]	[46] [48]			

Colour code:

Period 1 (pre 1950s - 1978)
Period 2 (1978 - 1982)
Period 3 (1982 - 2000)
Period 4 (2000 -)

References:

1. Fitzpatrick, J. and W.Y. Ako, *Empowering the initiation of a prevention strategy to combat malaria in Papua New Guinea.* Rural Remote Health, 2007. **7**(2): p. 693.
2. Shiff, C., *Vector control community participation and malaria morbidity in the Bagamoyo District of rural Tanzania.* Research and Reviews in Parasitology, 1998. **58**(3/4): p. 209-215.
3. Riji, H.M., *Adopting the primary health care approach in malaria control in Malaysia: lessons in community participation.* Southeast Asian Journal of Tropical Medicine and Public Health, 1992. **23**(Suppl. 1): p. 18-22.
4. Mantra, I.B., *The role of community participation in the malaria control program in Indonesia.* Southeast Asian J Trop Med Public Health, 1992. **23 Suppl 1**: p. 23-9.
5. Silva, K.T., et al., *Malaria control through community action at the grass-roots: Experience of the Sarvodaya malaria control research project in Sri Lanka from 1980 to 1986.* 1988, TDR, World Health Organisation Geneva. p. 1-68.
6. Kaseje, D.C. and E.K. Sempebwa, *An integrated rural health project in Saradidi, Kenya.* Soc Sci Med, 1989. **28**(10): p. 1063-71.
7. Okanurak, K. and S. Sornmani, *Community participation in the malaria control program in Thailand: a review.* Southeast Asian J Trop Med Public Health, 1992. **23 Suppl 1**: p. 36-43.
8. Delacollette, C., P. Van der Stuyft, and K. Molima, *Using community health workers for malaria control: experience in Zaire.* Bull World Health Organ, 1996. **74**(4): p. 423-30.
9. Rojas, W., S. Botero, and H.I. Garcia, *An integrated malaria control program with community participation on the Pacific Coast of Colombia.* Cad Saude Publica, 2001. **17 Suppl**: p. 103-13.
10. Garfield, R.M. and S.H. Vermund, *Health education and community participation in mass drug administration for malaria in Nicaragua.* Soc Sci Med, 1986. **22**(8): p. 869-77.
11. Ghebreyesus, T.A., et al., *Community participation in malaria control in Tigray region Ethiopia.* Acta Trop, 1996. **61**(2): p. 145-56.
12. Rajagopalan, P.K. and K.N. Panicker, *Feasibility of community participation for vector control in villages.* Indian J Med Res, 1984. **80**: p. 117-24.
13. Sharma, V.P., *Community-based malaria control in India.* Parasitol Today, 1987. **3**(7): p. 222-6.
14. Castro, M.C., et al., *Community-based environmental management for malaria control: evidence from a small-scale intervention in Dar es Salaam, Tanzania.* Malar J, 2009. **8**: p. 57.
15. Azhar, M., et al., *Participatory disease surveillance and response in Indonesia: strengthening veterinary services and empowering communities to prevent and control highly pathogenic avian influenza.* Avian Dis, 2010. **54**(1 Suppl): p. 749-53.
16. Babu, B.V., et al., *Use of an inclusive-partnership strategy in urban areas of Orissa, India, to increase compliance in a mass drug administration for the control of lymphatic filariasis.* Ann Trop Med Parasitol, 2006. **100**(7): p. 621-30.
17. Harkins, T., et al., *The health benefits of social mobilization: experiences with community-based Integrated Management of Childhood Illness in Chao, Peru and San Luis, Honduras.* Promot Educ, 2008. **15**(2): p. 15-20.
18. Malecela, M.N., et al., *The sharp end - experiences from the Tanzanian programme for the elimination of lymphatic filariasis: notes from the end of the road.* Ann Trop Med Parasitol, 2009. **103 Suppl 1**: p. S53-7.
19. Katabarwa, M.N., et al., *Traditional kinship system enhanced classic community-directed treatment with ivermectin (CDTI) for onchocerciasis control in Uganda.* Trans R Soc Trop Med Hyg, 2010. **104**(4): p. 265-72.
20. Ramaiah, K.D., et al., *Effectiveness of community and health services-organized drug delivery strategies for elimination of lymphatic filariasis in rural areas of Tamil Nadu, India.* Trop Med Int Health, 2001. **6**(12): p. 1062-9.
21. Hoy, D., et al., *Building capacity and community resilience to HIV: a project designed, implemented, and evaluated by young Lao people.* Glob Public Health, 2008. **3**(1): p. 47-61.
22. Perez, D., et al., *Community participation in Aedes aegypti control: a sociological perspective on five years of research in the health area ''26 de Julio'', Havana, Cuba.* Trop Med Int Health, 2007. **12**(5): p. 664-72.
23. Guthmann, J.P., et al., *Patients' associations and the control of leishmaniasis in Peru.* Bull World Health Organ, 1997. **75**(1): p. 39-44.
24. Cline, B.L. and B.S. Hewlett, *Community-based approach to schistosomiasis control.* Acta Trop, 1996. **61**(2): p. 107-19.
25. Katsivo, M.N., et al., *Involvement of a community in schistosomiasis control: a Kenyan experience.* East Afr Med J, 1993. **70**(8): p. 478-81.
26. Isely, R.B., *Reflections on an experience in community participation in Cameroon.* Annales de la Societe Belge de Medicine Tropicale, 1979. **59**(Suppl): p. 103-115.
27. Chandiwana, S.K., P. Taylor, and D. Matanhire, *Community control of schistosomiasis in Zimbabwe.* Cent Afr J Med, 1991. **37**(3): p. 69-77.
28. Bryan, R.T., et al., *Community participation in vector control: lessons from Chagas' disease.* Am J Trop Med Hyg, 1994. **50**(6 Suppl): p. 61-71.
29. Toledo, M.E., et al., *Towards active community participation in dengue vector control: results from action research in Santiago de Cuba, Cuba.* Trans R Soc Trop Med Hyg, 2007. **101**(1): p. 56-63.
30. Loue, S., L.S. Lloyd, and E. Phoombour, *Organising Asian Pacific Islanders in an urban community to reduce HIV risk: a case study.* AIDS Educ Prev, 1996. **8**(5): p. 381-393.
31. Kassambara, M., et al., *Village community participation in onchocerciasis vector control* World Health Forum, 1986. **7**(1): p. 57-61.
32. Boelee, E. and H. Laamrani, *Environmental control of schistosomiasis through community participation in a Moroccan oasis.* Tropical Medicine & International Health, 2004. **9**(9): p. 997-1004.
33. Khun, S. and L. Manderson, *Community participation and social engagement in the prevention and control of dengue fever in rural Cambodia.* Dengue Bulletin, 2008. **32**: p. 145-155.
34. Magnussen, P., et al., *The impact of a school health programme on the prevalence and morbidity of urinary schistosomiasis in Mwera Division, Pangani District, Tanzania.* Trans R Soc Trop Med Hyg, 2001. **95**(1): p. 58-64.
35. CDI Study Group, *Community-directed interventions for priority health problems in Africa: results of a multicountry study.* Bull World Health Organ, 2010. **88**(7): p. 509-18.
36. Ndekha, A., et al., *Community participation as an interactive learning process: experiences from a schistosomiasis control project in Zimbabwe.* Acta Tropica, 2003. **85**(3): p. 325-338.
37. Gurtler, R.E., et al., *Sustainable vector control and management of Chagas disease in the Gran Chaco, Argentina.* Proc Natl Acad Sci U S A, 2007. **104**(41): p. 16194-9.
38. Okonofua, F.E., et al., *Impact of an intervention to improve treatment-seeking behavior and prevent sexually transmitted diseases among Nigerian youths.* Int J Infect Dis, 2003. **7**(1): p. 61-73.
39. Nathan, M.B., L. Lloyd, and A. Wiltshire, *Community participation in environmental management for dengue vector control: experiences from the English-speaking Caribbean.* Dengue Bulletin, 2004. **28**(Supplement): p. 13-16.
40. Panicker, K.N. and V. Dhanda, *Community participation in the control of filariasis.* World Health Forum, 1992. **13**(2-3): p. 177-81.
41. Garcia-Zapata, M.T. and P.D. Marsden, *Chagas' disease: control and surveillance through use of insecticides and community participation in Mambai, Goias, Brazil.* Bull Pan Am Health Organ, 1993. **27**(3): p. 265-79.
42. Kaneko, A., *A community-directed strategy for sustainable malaria elimination on islands: Short-term MDA integrated with ITNs and robust surveillance.* Acta Trop, 2010.
43. Chen, W.I., *Malaria eradication in Taiwan, 1952-1964--some memorable facts.* Gaoxiong Yi Xue Ke Xue Za Zhi, 1991. **7**(5): p. 263-70.
44. Sleigh, A., et al., *Eradication of schistosomiasis in Guangxi, China. Part 1: Setting, strategies, operations, and outcomes, 1953-92.* Bulletin of the World Health Organization, 1998. **76**(4): p. 361-372.
45. Obregon, R., et al., *Achieving polio eradication: a review of health communication evidence and lessons learned in India and Pakistan.* Bull World Health Organ, 2009. **87**(8): p. 624-30.
46. Edungbola, L.D., et al., *Mobilization strategy for guinea worm eradication in Nigeria.* Am J Trop Med Hyg, 1992. **47**(5): p. 529-38.
47. Foster, S.O., *Participation of the public in global smallpox eradication.* Public Health Rep, 1978. **93**(2): p. 147-9.
48. Sam-Abbenyi, A., et al., *Dracunculiasis in Cameroon at the threshold of elimination.* Int J Epidemiol, 1999. **28**(1): p. 163-8.
49. WHO, *The global eradication of smallpox: final report of the global commission for the certification of smallpox eradication*. 1980, Geneva: World Health Organisation. 122.
50. Hii, J.L., et al., *Sustainability of a successful malaria surveillance and treatment program in a Runggus community in Sabah, east Malaysia.* Southeast Asian J Trop Med Public Health, 1996. **27**(3): p. 512-21.
51. Mutalemwa, P., et al., *Community directed approach beyond ivermectin in Tanzania: a promising mechanism for the delivery of complex health interventions.* Tanzan J Health Res, 2009. **11**(3): p. 116-25.
52. Sanchez, L., et al., *Intersectoral coordination, community empowerment and dengue prevention: six years of controlled interventions in Playa Municipality, Havana, Cuba.* Trop Med Int Health, 2009. **14**(11): p. 1356-64.
53. Constantinou, K., *Anopheles (malaria) eradication in Cyprus.* Parassitologia, 1998. **40**(1-2): p. 131-5.
54. Ruebush, T.K., 2nd and H.A. Godoy, *Community participation in malaria surveillance and treatment. I. The Volunteer Collaborator Network of Guatemala.* Am J Trop Med Hyg, 1992. **46**(3): p. 248-60.
55. Jacobs, B. and N. Price, *Community participation in externally funded health projects: lessons from Cambodia.* Health Policy Plan, 2003. **18**(4): p. 399-410.
56. Walt, G., M. Perera, and K. Heggenhougen, *Are large-scale volunteer community health worker programmes feasible? The case of Sri Lanka.* Social Science and Medicine, 1989. **29**(5): p. 599 - 608.
57. Richards, F., Jr., et al., *Community-based ivermectin distributors: onchocerciasis control at the village level in Plateau State, Nigeria.* Acta Trop, 1996. **61**(2): p. 137-44.
58. Kironde, S. and M. Kahirimbanyi, *Community participation in primary health care (PHC) programmes: lessons from tuberculosis treatment delivery in South Africa.* Afr Health Sci, 2002. **2**(1): p. 16-23.
59. Lin-hua, T., Q. Hui-lin, and X. Shu-hui, *Malaria and its control in the People's Republic of China.* Southeast Asean Journal of Tropical Medicine and Public Health, 1991. **22**(4): p. 467-476.
60. Garfield, R., *Malaria control in Nicaragua: social and political influences on disease transmission and control activities.* Lancet, 1999. **354**(9176): p. 414-8.
