# Supplementary material for: The architecture and effect of participation: a systematic review of community participation for communicable disease control and elimination. Implications for malaria elimination
Source: Malar J. 2011 Aug 4;10:225. doi: 10.1186/1475-2875-10-225 (PMC3171376; doi:10.1186/1475-2875-10-225)
Supplement: Additional file 3 — Summary of the quantitative evidence: study characteristics and selected results. [file 1475-2875-10-225-S3.PDF]

Additional file 3 – Summary of the quantitative evidence: study characteristics and selected results.

Reference	Study location	Intervention			Control		Results relating to main indicator/s
		Unit of allocation	Target groups	Intervention	Unit of allocation	Specification of the control	
[a]Okonofua et. al. (2003)	Benin City, Nigeria	Primary schools (urban area)	Randomly selected adolescents in senior classes at 4 schools (ages 14-20yrs) *n*=643	• Use of educational meetings and participatory activities via a 'reproductive health club'<br>• Training and use of peer educators<br>• Training of healthcare providers	Two control group - random selection of 4 schools in Benin City (C1) *n*=649 and 4 schools in Ekpoma (C2) *n*=604	No intervention	Statistically significant reduction in prevalence of STD symptoms in intervention group compared to C1 (OR=0.63, 95%CI=0.43-0.91) and C2 (OR=0.69, 95%CI=0.48-0.98).
[a]Kironde & Kahirimbanyi (2002)	Northern Cape Province, South Africa	45 randomly selected Primary Health Care Facilities	Patients with TB (*n*=769)	• Patient selected treatment option (clinic or home-based DOT* or self-administration)<br>• Training and use of community based volunteers for DOT.<br>• Adequate supervision of volunteers	n/a	n/a	Treatment outcomes for community-based DOT* just as effective other treatment modes (RR=1.04[0.94-1.16], p=0.435) for new patients & superior to self-administration for re-treatment patients (RR=5.89[2.3-15.9], p<0.001).
[a]Delacollette et al. (1996)	Katana, Zaire	Katana health zone - 12 villages in Area A (approx 1500 population)	Area A (patients presenting to community based volunteer with fever over study period; *n*=484)	• Use of educational messages<br>• Community involvement in planning / implementation<br>• Use of local volunteers for presumptive malaria treatment & education<br>• Training & symbolic monetary incentive	Katana health zone - Area B (approx 1500 population). Patients presenting with fever; *n*=471)	Malaria treatment at health centre only	Significant reduction in mean malaria incidence per 10,000 person-weeks in Area A compared to B. Rate ratio over 2 years: Area A – 1.9 (95%CI=1.7-2.2) Area B - 1.1 (95%CI=1.0-1.2)

[a]Hii et al. (1996)	Sabah, Malaysia	Kudat district, 13 villages (pop. 4950) in initial recruitment.	Patients presenting to Community Health Volunteer with fever (CP intervention area)	• Community selected health volunteers<br>• Training & supervision of volunteers to provide presumptive malaria treatment & taking blood films.	Non-participating villages in Kudat district (unclear) (non-CP areas)	Malaria treatment at health centres, district hospital and flying doctor service as per routine health care protocols.	Annual *P. vivax* & *P. falciparum* positivity rates were significantly higher in non-CP areas than in CP villages (p<0.05) (with the exception of *P.falciparum* in the final study year). Malaria mortality did not differ between groups.
[b] Sanchez et al. (2009)	Havana, Cuba	Playa Municipality, CP4 area (population of 27,030); two differing intervention areas. Program extension to CP6 area (population of 16,096)	Assessment of community participation (Rifkin framework) plus questionnaire administered via systematic random sampling of households in 2 intervention areas at 3 time points (*n*=750).	• Engagement of multi-sectoral stakeholders / opinion leaders<br>• Use of tertiary education institution to carry out training<br>• Stakeholder training in situation analysis & fostering participation in strategic planning<br>• Use of community working groups<br>• Use of community empowerment approach in subset of villages<br>• Routine education and control activities	Playa Municipality, CP5 area (population of 14,219). Assessment of community participation using Rifkin framework	Routine education and control activities	In intervention area: Mean participation scores significantly higher than baseline (1.6) following intersectoral coordination phase (3.4) and empowerment phase (4.4). More than 80% of households improved participation in dengue prevention (source reduction & sanitation activities)<br>Statistically significant difference in entomological impact (Breteau Index) between control and intervention areas throughout study period.
[b]Toledo et al. (2007)	Santiago de Cuba, Cuba	Random selection of 20 neighbour-hoods from 3 health areas.	200 households randomly selected from neighbourhoods	• Community Working Group created by key stakeholders<br>• Assessment of learning needs followed by training.<br>• Needs identification	Random selection of 20 neighbour-hoods from 3 health areas.	Standard vertically applied control activities, education and enforcement of vector control legislation	Pre-intervention participation scores in 3 neighbourhoods were; 2, 1.6 and 1.2. Post-intervention scores increased to 4.4, 4.4, 2.2 based on Rifkin tool. [120]

				and intersectoral actions at local level<br>• Support from tertiary education institution<br>• Provision of adequate resources<br>• Communication strategy developed<br>• Interpersonal education, mass media & community meetings<br>• Risk surveillance through participatory mapping		through penalties	Potential mosquito breeding sources decreased by 46.7% (p<0.01).<br>Acceptability of larvicide increased from 54.5% to 99% (p<0.01).<br>Entomological indicators - similar reductions b/w control and intervention groups but longer term follow-up revealed lack of sustainability of reductions in control group.[119]
[b]Castro et al. (2009)	Dar es Salaam, Tanzania	Purposeful selection of 2 communities. Environmental Modification (EM = drain clearing) plus community participation.	Repeated surveys in 75 randomly selected households (2 adults & 2 children) in each community	• Partnership b/w NGO & health authority<br>• Community involved with planning drain clearing activities<br>• Employment of locals for activities<br>• SOPs developed<br>• Community sensitization (community leader seminars, mass meetings, household visits)<br>• Maintenance phase responsibility & resources transferred to communities	4 purposefully selected communities similar in characteristics to intervention group. Repeated surveys in 75 randomly selected households in each community (2 adults & 2 children per household).	2 communities larviciding in drains (LV)<br>2 communities with no intervention (NO)	Comparison of effect of community participation on disease control limited by confounding effects of 3 different control interventions.<br>Community perceptions of benefits of drain cleaning significantly higher in EM intervention group (61%) than LVC group (30%).<br>Despite high voluntary participation in initial cleaning (in EM group), this was not sustained in maintenance phase due to lack of financial incentives.
[b]CDI study group (2010)	Cameroon, Nigeria & Uganda, Africa	7 research sites, 4 districts at each site	10 villages randomly selected from each district. All	• Stakeholder engagement<br>• Participatory process with communities	7 research sites, 1 district at each site allocated to control (7	Conventional non-integrated delivery of the 5 interventions.	Coverage for vitamin A supplementation, insecticide-treated nets and anti-malaria treatment

		allocated to intervention (28 districts) Progressive integration of 5 community directed PHC interventions (CDI).	eligible household members of 5 households then randomly selected from each village for survey.	directing planning, implementation & monitoring of interventions.<br>• Volunteers selected by communities & trained / supervised by health workers.<br>• Community determined incentives for volunteers.<br>• Resources provided by health authority.	districts)		significantly higher when delivered through the CDI process (p<0.001).<br>No sig. difference b/w CDI and control districts with directly-observed treatment.<br>Ivermectin coverage 10% higher in districts where multiple interventions were delivered through CDI approach.
[b]Ramaiah et al. (2001)	Tamil Nadu, India	Randomly selected villages (20) stratified by presence or absence of health facility.	Each member of randomly selected households (20) from each of the 20 villages.	• Drug distribution entirely devolved to communities (ComDT arm); including timing , duration and mode of distribution of drug; selection of distributors and record keeping.<br>• Education and community meetings<br>• Minimal role of health staff except in engagement of community leaders and training of distribution volunteers.<br>• House-to-house drug delivery	Randomly selected villages (20) stratified by presence or absence of health facility.	• Drug distribution carried out by Ministry of Health staff through the PHC system under supervision by medical officers (HST arm).<br>• Medical officers trained healthcare workers to distribute drug<br>• House-to-house drug delivery	Drug distribution - No significant difference between ComDT & HST arms (66% & 74% respectively; p>0.05).<br>Drug consumption – No significant difference between ComDT & HST arms (53% & 59% respectively; p>0.05).<br>Both ComDT & HST arms had poor compliance with drug consumption.
[b]Jacobs & Price (2003)	Maung Russay & Kirivong	Two randomly selected villages per	Committee members and women with	• MoH vehicles for community participation in	18 randomly selected villages in Kirivong	• Engagement of existing Pagoda	At Maung Russay – 69% of reps reported they were active (*n*=32).

	Districts, Cambodia	health centre in Maung Russay District (20 villages) with Health Centre Co-Management Committees (HCCMCs) and Feedback Committees (FBCs)	children <5yrs of age (randomly selected) were surveyed.	planning and implementation of health services introduced<br>• HCCMC – 3 health centre staff, 2 elected community reps from each commune.<br>• FBC – HCCMC plus one male & one female rep from each village<br>• Community selected the representatives	District (9 with & 9 without Buddist pagoda committees).	committee – 5 to 7 members including the abbot, elected monks & respected community elders.<br>• Reps to the local HCCMC & FBC committees selected by Chief monk (one male & one female).	At Kirivong – all reps reported they were active (*n*=46).<br>At Maung Russay – 62% of women with children <5yrs reported they knew a committee member; 78% of those said they would disclose a physical problem & 29% a personal problem.<br>At Kirivong – 63% of women reported they knew a committee member; 92% of those reported they would disclose a physical problem & 67% a personal problem.<br>Engagement of existing community-based structures more effective for community participation than externally introduced structures.
[b]Katabarwa et al. (2010)	Hoima & Moyo Districts, Uganda	Three sub-counties of Moyo District randomly selected;15 communities from these sub-counties randomly selected.	Interviews with a male & female member of each of 15 households in each community (*n*=447).<br>Interviews also with community distributors.	• Kinship enhanced Community Directed Intervention (CDI) approach.<br>• Engagement of traditional kinship systems<br>• Kinship zones identified by community members<br>• Each zone selects its own community distributors,	Four sub-counties of Hoima District randomly selected; 25 communities randomly selected.<br>Interviews with 15 households in each community (*n*=750).<br>Interviews also	• Classic CDI approach<br>• Reps render services to all community members irrespective of kinships.<br>• Reps supervised by health workers rather than community	Overall treatment coverage in classic CDI group was 76.4% in 2005 and dropped to 62.1% in 2006.<br>In kinship enhanced CDI, treatment coverage maintained at 93.7% for both years.<br>In classic CDI 50.8% of community leaders controlled where treatment centres would be located compared to

				supervisors, methods of treatment, health education & training centres.<br>• Reps act only within their kinship zone	with community distributors	supervisors	6.8% in kinship enhanced CDI (p<0.001).<br>There was significantly better disease knowledge in the kinship enhanced CDI group than classic CDI group.
[b]Babu et al. (2006)	Orissa, India	17 wards sampled in urban Choudwar.	Cluster randomised selection of participating households in each ward (*n*=850)	• Formative research to identify sub-groups at risk of marginalization and inclusion of these groups as stakeholders.<br>• Stakeholder involvement in MDA planning & decision making.<br>• Engagement of ward level partners in micro-level planning.<br>• Volunteer distributors elected by ward partners.<br>• IEC materials, mass media, house-to-house visits & school rallies.	Dhenkanal (urban area) – 6 wards purposefully selected to cover all SES strata. Cluster randomised selection of households (*n*=180).<br>Tangi PHC (rural area) – 6 villages randomly selected. Cluster randomised selection of households (*n*=150).	• MDA distributed through standard PHC services.	Household coverage of MDA significantly higher in urban intervention community (93.7%) compared to urban control community (73%), but similar to rural PHC village (97.8%).<br>Large gap between coverage and compliance in all 3 communities but non-compliance greater in Urban control (Dhenkanal).

[a] Contributes to evidence on the effectiveness of community participation for disease control / elimination
[b] Contributes to evidence on the effectiveness of the various community participation strategies on the level of participation achieved
*Directly Observed Treatment
