# Supplementary material for: The architecture and effect of participation: a systematic review of community participation for communicable disease control and elimination. Implications for malaria elimination
Source: Malar J. 2011 Aug 4;10:225. doi: 10.1186/1475-2875-10-225 (PMC3171376; doi:10.1186/1475-2875-10-225)
Supplement: Additional file 4 — Summary of lessons learned from quantitative research papers. [file 1475-2875-10-225-S4.PDF]

Additional file 4 – Summary of lessons learned from quantitative research papers examining the effectiveness of community participation for disease control / elimination.

Reference	Location of study	Disease addressed	Intervention	Lessons learned
Okonofua et. al. (2003)	Benin City, Nigeria	Sexually transmitted diseases	• Use of educational meetings and participatory activities via a 'reproductive health club'<br>• Training and use of peer educators<br>• Training of healthcare providers	• Peer education and counselling on STDs to school adolescents significantly improves care-seeking behaviours and reduces episodes of STDs.<br>• Intervention did not alter strong belief in traditional STD treatment; hence increase in use of traditional healers post intervention.<br>• The intervention did not improve condom use due to their lack of acceptability.<br>• Targeting schools for reproductive health programs can be expected to reach a high proportion of adolescents in Nigeria.
Kironde & Kahirimbanyi (2002)	Northern Cape Province, South Africa	TB	• Patient selected treatment option (clinic or home-based DOT* or self-administration)<br>• Training and use of community based volunteers for DOT.<br>• Adequate supervision of volunteers	• Lay volunteers can effectively administer low technology interventions and provide a viable supplementary option to other treatment delivery modes.<br>• They provide a cost-effective delivery mechanism.<br>• Lack of monetary incentive can lead to high attrition rates of volunteers particularly in areas of high unemployment.<br>• There were gender differences in those willing to volunteer without incentive (majority were women).
Delacollette et al. (1996)	Katana, Zaire	Malaria	• Use of educational messages<br>• Community involvement in planning / implementation<br>• Use of local volunteers for presumptive malaria treatment & education<br>• Training & symbolic monetary incentive	• Significant reduction in mean malaria incidence in intervention group resulted from the presence of malaria dedicated CHWs providing increased access to timely and appropriate treatment.<br>• Low education level, high female illiteracy, non-comprehensiveness of CHW care and perceived appropriateness of home treatment compromise the sustainability of the use of CHWs.<br>• Intervention had no effect on malaria mortality.
Hii et al. (1996)	Sabah, Malaysia	Malaria	• Community selected health volunteers<br>• Training & supervision of volunteers to provide presumptive malaria treatment & taking of blood films.	• Success attributed to ability of volunteer health workers to access remote communities as well as their adequate supervision in the field by VBDCP staff.<br>• Scale up of use of volunteers is hindered by inadequacies in health system infrastructure.<br>• Efficiency of disease specific volunteer health workers is compromised in low transmission areas.<br>• Intervention had no effect on malaria mortality.

Summary of lessons learned from quantitative research papers examining the effectiveness of community participation strategies on the level of intervention coverage and / or participation achieved.

Reference	Location of study	Disease addressed	Intervention	Lessons learned
Sanchez et al. (2009)	Havana, Cuba	Dengue	• Engagement of multi-sectoral stakeholders<br>• Tertiary education institution carried out training<br>• Stakeholder training in situation analysis & fostering participation in strategic planning<br>• Use of community working groups<br>• Use of community empowerment approach in subset of villages<br>• Education activities	• Approach increased local capacity for self reflection and to generate participation for dengue prevention<br>• Combination of community empowerment and mulit-sectoral collaboration resulted in sustainability of program following withdrawal of external support<br>• Importance of integrating dengue control with community priorities such as improved sanitation.
Toledo et al. (2007)	Santiago de Cuba, Cuba	Dengue	• Community Working Group created by key stakeholders<br>• Assessment of learning needs followed by training.<br>• Needs identification and intersectoral actions at local level<br>• Support from tertiary education institution<br>• Provision of adequate resources<br>• Communication strategy developed<br>• Interpersonal education, mass media & community meetings<br>• Risk surveillance through participatory mapping	• Affective vector control achieved and significant reduction in environmental risks sustained over the longer-term in intervention group only<br>• Success attributed to behaviour change resulting from local identification of problems & needs; a process supported by the key stakeholder groups<br>• Education alone is insufficient<br>• Community-local government partnership important for developing and sustaining the capacity to resolve problems of mutual concern<br>• The importance of the community as an actor in the program rather than just a passive recipient is highlighted
Castro et al. (2009)	Dar es Salaam, Tanzania	Malaria	• Partnership b/w NGO & health authority<br>• Community involved with planning drain clearing activities<br>• Employment of locals for activities<br>• SOPs developed<br>• Community sensitization (community leader seminars, mass meetings, household visits)	• Ongoing individual participation in environmental management without incentive is not feasible<br>• Lack of intersectoral collaboration contributed to unsuccessful maintenance of activities in one community.<br>• Critical to sustaining participation and fostering intersectoral linkages is political commitment, the building of local capacity & provision of sufficient resources.

			• Maintenance phase responsibility & resources transferred to communities	• Community sensitisation / education activities need to be conducted on a regular basis to maintain momentum for participation.
CDI study group (2010)	Cameroon, Nigeria & Uganda, Africa	Onchocerciasis / integrated PHC	• Stakeholder engagement<br>• Participatory process with communities directing the planning, implementation & monitoring of interventions.<br>• Volunteers selected by communities & trained / supervised by health workers.<br>• Community determined incentives for volunteers.<br>• Resources provided by health authority.	• Benefits of integrated CDI approach:<br>▪ Provides effective platform for integrated and sustained delivery of PHC interventions of various complexities.<br>▪ cost savings achieved with delivery and implementation of integrated interventions<br>▪ Increased community awareness of health rights and participation<br>▪ Empowerment of women<br>• Difficulties encountered:<br>▪ As CDI approach is embedded in health system it is subject to health system constraints<br>▪ Acceptability of interventions still a barrier in some areas<br>▪ Buy-in to the CDI approach by communities, health workers, policy-makers & other stakeholders takes time.
Ramaiah et al. (2001)	Tamil Nadu, India	Lymphatic filariasis	• Drug distribution entirely devolved to communities including timing, duration and mode of distribution of drug, selection of distributors and record keeping.<br>• Education and community meetings<br>• Minimal role of health staff except in engagement of community leaders and training of distribution volunteers.<br>• House-to-house drug delivery	• Demonstrated that prophylactic drug delivery of DEC for lymphatic filariasis can be entrusted to communities and does not require close involvement of medical personnel.<br>• Barriers to community directed treatment;<br>▪ Poor knowledge of the disease<br>▪ Poor response by community leaders<br>▪ Lack of intervention acceptability<br>▪ Group and cast conflicts<br>▪ Alternative community health & disease priorities<br>▪ Insufficient human resources
Jacobs & Price (2003)	Maung Russay & Kirivong Districts, Cambodia	General Primary health care services	• Externally introduced systems for community participation in planning and implementation of health services (Health Centre Co-management committee; HCCMC & feedback committee; FBC) *vs.* use of existing community systems (pagoda committee)<br>• Community selected representatives *vs.* representatives appointed by pagoda chief	• Highlights the importance of selecting correct community representatives as the acceptability of these representatives by the community affects their participation in disease control measures.<br>• Engagement of existing community-based structures is more effective for community participation than externally introduced structures particularly for externally funded health projects with short implementation timeframes. Existing

			monks.	mechanisms often have the advantage of having established and trusted leadership, local organization, resource mobilisation and management.<br>• Participatory research advocated as important for identifying the most appropriate local organisation to lead initiatives.<br>• Democratic election of community representatives may not be essential for community participation in countries that have experienced long standing authoritarian governance.
Katabarwa et al. (2010)	Hoima & Moyo Districts, Uganda	Onchocerciasis	• Kinship enhanced Community Directed Intervention approach.<br>• Engagement of traditional kinship systems<br>• Kinship zones identified by community members<br>• Each zone selects its own community distributors, supervisors, methods of treatment, health education & training centres.<br>• Distributors act only within their kinship zone	• Kinship enhanced CDI approach was superior to the classic CDI approach in terms of treatment coverage, reduced decision making by community leaders, mobilisation for CDI activities, enhanced participation of women and reduced attrition of distributors and reduced demands for incentives.<br>• Community participation for health interventions more likely to be sustainable along kinship lines than administrative boundaries in traditional communities.<br>• Kinship enhanced CDI produced greater numbers of distributors, reducing the workload of each and providing the potential for involvement in other health activities.
Babu et al. (2006)	Orissa, India	Lymphatic filariasis	• Formative research to identify sub-groups at risk of marginalization and inclusion of these groups as stakeholders.<br>• Stakeholder involvement in MDA planning & decision making (local govt., public & private health workers, youth & women's groups, residents' clubs, local industry reps., prisons, schools, religious & ethnic groups).<br>• Engagement of urban ward-level partners in micro-planning.<br>• Volunteer distributors elected by ward partners & provided training & supervision.<br>• Education materials, mass media, house-to-house visits & school rallies carried out.	• Community participation important for achieving significantly higher household coverage of MDA in urban areas.<br>• Compliance remains a problem despite community participation strategy due to lack of intervention acceptability (particularly in young children).<br>• Relative affluence of urban communities leads to greater access of private sector for health needs compared to rural communities and hence it is important to engage the private sector.
